# Machine learning-assisted identification of bioindicators predicts medium-chain carboxylate production performance of an anaerobic mixed culture

**DOI:** 10.1186/s40168-021-01219-2

**Published:** 2022-03-25

**Authors:** Bin Liu, Heike Sträuber, João Saraiva, Hauke Harms, Sandra Godinho Silva, Jonas Coelho Kasmanas, Sabine Kleinsteuber, Ulisses Nunes da Rocha

**Affiliations:** 1 Department of Environmental Microbiology, Helmholtz Centre for Environmental Research – UFZ, Leipzig, Germany; 2 Institute for Bioengineering and Biosciences, Department of Bioengineering, Instituto Superior Técnico Universidade de Lisboa, Lisbon, Portugal; 3 Institute of Mathematics and Computer Sciences, University of São Paulo, São Carlos, Brazil; 4 Department of Computer Science and Interdisciplinary Center of Bioinformatics, University of Leipzig, Leipzig, Germany

**Keywords:** Predictive biology, Carboxylate platform, Model ecosystems, Reactor microbiota, Microbial chain elongation

## Abstract

**Background:**

The ability to quantitatively predict ecophysiological functions of microbial communities provides an important step to engineer microbiota for desired functions related to specific biochemical conversions. Here, we present the quantitative prediction of medium-chain carboxylate production in two continuous anaerobic bioreactors from 16S rRNA gene dynamics in enriched communities.

**Results:**

By progressively shortening the hydraulic retention time (HRT) from 8 to 2 days with different temporal schemes in two bioreactors operated for 211 days, we achieved higher productivities and yields of the target products *n*-caproate and *n*-caprylate. The datasets generated from each bioreactor were applied independently for training and testing machine learning algorithms using 16S rRNA genes to predict *n*-caproate and *n*-caprylate productivities. Our dataset consisted of 14 and 40 samples from HRT of 8 and 2 days, respectively. Because of the size and balance of our dataset, we compared linear regression, support vector machine and random forest regression algorithms using the original and balanced datasets generated using synthetic minority oversampling. Further, we performed cross-validation to estimate model stability. The random forest regression was the best algorithm producing more consistent results with median of error rates below 8%. More than 90% accuracy in the prediction of *n*-caproate and *n*-caprylate productivities was achieved. Four inferred bioindicators belonging to the genera *Olsenella*, *Lactobacillus*, *Syntrophococcus* and *Clostridium* IV suggest their relevance to the higher carboxylate productivity at shorter HRT. The recovery of metagenome-assembled genomes of these bioindicators confirmed their genetic potential to perform key steps of medium-chain carboxylate production.

**Conclusions:**

Shortening the hydraulic retention time of the continuous bioreactor systems allows to shape the communities with desired chain elongation functions. Using machine learning, we demonstrated that 16S rRNA amplicon sequencing data can be used to predict bioreactor process performance quantitatively and accurately. Characterizing and harnessing bioindicators holds promise to manage reactor microbiota towards selection of the target processes. Our mathematical framework is transferrable to other ecosystem processes and microbial systems where community dynamics is linked to key functions. The general methodology used here can be adapted to data types of other functional categories such as genes, transcripts, proteins or metabolites.

Video Abstract

**Supplementary Information:**

The online version contains supplementary material available at 10.1186/s40168-021-01219-2.

## Background

Microbes form complex communities that play essential roles in ecosystem functioning. Identifying bioindicators derived from community analysis and using them to predict process performance may delineate potential cause-effect relationships with ecosystem functioning [1, 2]. The knowledge gained from prediction can be used to generate hypotheses on the role of key species. At the ecosystem level, designing effective control strategies for key species holds promise to manage the community towards the selection of the target processes, which is crucial for microbiota-based biotechnologies [3–5].

Our goals were to investigate how environmental manipulations affect ecosystem functioning and to predict performance metrics of the quantifiable biological processes by following microbial community dynamics. Model ecosystems offer the opportunity to link microbial diversity and ecosystem functioning in a quantifiable and predictable way [6–8]. Such simplified ecosystems can still be complex regarding microbial interactions and involved metabolic processes [6]. Here, we used anaerobic fermentation reactors as model ecosystems and considered microbial chain elongation (CE) as the quantifiable model ecosystem process. CE is a microbial process that produces medium-chain carboxylates (6 to 8 carbon atoms) through reverse β-oxidation [9]. Recently we enriched a mixed culture that produces *n*-butyrate (C4), *n*-caproate (C6) and *n*-caprylate (C8) from xylan and lactate in a daily-fed reactor system [10], to simulate the feedstock conditions of anaerobic fermentation of ensiled plant biomass [11]. For this bioprocess to be viable, it needs to include diverse functions such as xylan hydrolysis, xylose fermentation and CE with lactate as electron donor. Mixed culture fermentation is characterized by different trophic groups that may cooperate or compete with each other to metabolize complex substrates [9]. Species involved in these interactions can drive shifts in community structure and function [1]. During the long-term stable reactor operation, the community developed towards predominating C4 and biomass production at the cost of C6/C8 production [10]. The current study was conducted on the enriched chain-elongating microbiota in two parallel bioreactors to explore how process parameter changes shape the existing microbiota to optimise the process towards the target products C6 and C8. To promote C6 and C8 production and enrich the functional groups relevant to process performance, we reduced the hydraulic retention time (HRT). HRT refers to the average time soluble compounds reside in the bioreactor. Shortening the HRT is a common operation-based strategy for increasing C6/C8 production [12–16] and a key factor influencing microbial diversity [17]. It is relevant to the microbial growth rate in reactors without biomass retention, and it affects biomass concentration and community composition [18]. Following variations in diversity induced by HRT reduction, we tested if productivity and yield of the target products (C6 and C8) could be predicted by using machine learning. To provide insight into the community structure and function dynamics, we measured process performance and collected samples for community analysis using high-throughput sequencing of the 16S rRNA gene. Community analysis using 16S rRNA amplicon sequencing data combined with environmental variables can reveal relationships between microbial communities and ecosystem functioning. For example, Werner et al. demonstrated strong relationships between the phylogenetic community structure, reflected by time-resolved 16S rRNA amplicon data, and the methanogenic activity in full-scale anaerobic digesters, by applying constrained ordination [19].

Predictive analytics using machine learning has shown promise in microbiota-based biotechnologies [6, 20, 21]. The identification of bioindicators based on microbial community data is an important application of machine learning predictive models [22]. Different machine learning algorithms, such as linear regression [23], support vector machine [24] and random forest regression [25] have been used in microbiome studies. Our machine learning analysis consisted of the identification of the amplicon sequence variants (ASVs) that were relevant to community dynamics caused by HRT reduction and the prediction of C6/C8 production based on the selected ASVs (hereafter, HRT bioindicators). To determine the HRT bioindicators heuristically, we used the ASVs as features to predict the target HRT. We first used the microbiome automated machine learning pipeline (hereafter, mAML) [26] to test several different algorithms on our dataset for microbiome-based classification tasks. Once we had prediction accuracies from the different tested algorithms, we selected the algorithm with the highest prediction accuracy that can rank feature relevance. Since we want to gain insight into our data via the learned relationship between feature and target variable, it is crucial that the selected algorithm for suggesting bioindicators demonstrates not only high prediction accuracies but also is interpretable and can rank feature relevance. After determining the HRT bioindicators, we created C6/C8 production regression models using the selected ASVs. It is important to mention that our dataset is imbalanced regarding the number of samples from the different HRT. The dataset consists of 54 samples: 14 from HRT 8 days and 40 from HRT 2 days. Imbalanced datasets can create a bias to the learning task, prioritizing the prediction of the majority target. Consequently, to create the C6/C8 production regression model, we also determined the differences in the predictive performance of the original (unbalanced) datasets and of datasets that were balanced by oversampling to verify if our models can handle the imbalance found in our data. Finally, we used *k*-fold cross-validation to estimate the stability of the model.

## Results

### Effects of HRT decrease on process performance and microbial diversity

**Fig. 1:**
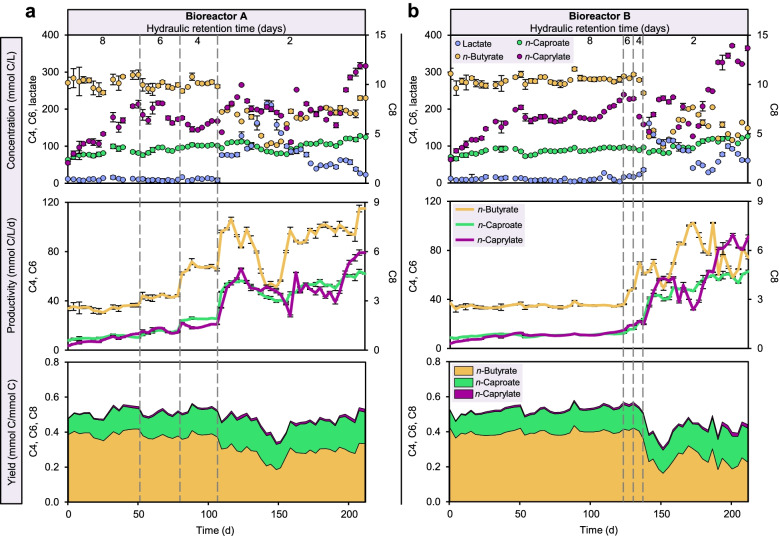
Performance of bioreactors. Concentrations of chain elongation products and lactate, as well as productivities and yields of chain elongation products in bioreactors A (**a**) and B (**b**) during the four HRT phases. Chain elongation products: C4, *n*-butyrate; C6, *n*-caproate; C8, *n*-caprylate

The progressive HRT decrease from 8 to 2 days increased the C6 and C8 productivities and yields in two independent bioreactors (Fig. 1). We first shortened the HRT to 6 days and then to 4 days in bioreactor A, which allowed the reactor microbiota to adapt to the new conditions and improved productivities of C4, C6 and C8 (Fig. 1a). Further HRT decrease to 2 days confirmed the increasing trend in productivity. At the end of the 2-day HRT period in bioreactor A, we achieved the highest productivities (mmol C L^−1^ day^−1^) of C4, C6 and C8 up to 115.0, 64.1 and 5.9, respectively. To confirm the observed effects of HRT shortening on the CE process and reactor microbiota, we executed a fast transition mode in bioreactor B and generated a different dataset from the parallel system. Comparable increases in productivity were observed (Fig. 1b). We obtained maximum productivities (mmol C L^−1^ day^−1^) of C4 up to 102.4, C6 up to 62.9 and C8 up to 7.0. The C6 and C8 yields (in terms of C mole product to consumed substrate ratio) increased along with decreasing HRT at the cost of C4 yield (Fig. 1 and Additional file 1: Table S1). Our results suggest that the shorter HRT favored lactate-based CE producing C6 and C8 over C4 production. C4 can be produced by CE of acetate and from sugars by butyric acid fermentation [27]. In both bioreactors at 2-day HRT, a temporary accumulation of lactate was observed that coincided with fluctuations of the C4, C6 and C8 production (Fig. 1a). Lactate concentrations were negatively correlated with C4 concentrations (Spearman’s rho = − 0.90, *P* < 0.05) and C6 concentrations (rho = − 0.89, *P* < 0.05), which reflects how lactate was produced and converted by the reactor microbiota. The HRT reduction resulted in higher gas production and hydrogen content (Additional file 1: Fig. S1). Besides, an increase in cell mass production (Additional file 1: Fig. S2) suggests a facilitating effect of short HRT on the growth of enriched populations with desirable activities; i.e., more biocatalysts were available in the high C6/C8 production phase.

**Fig. 2:**
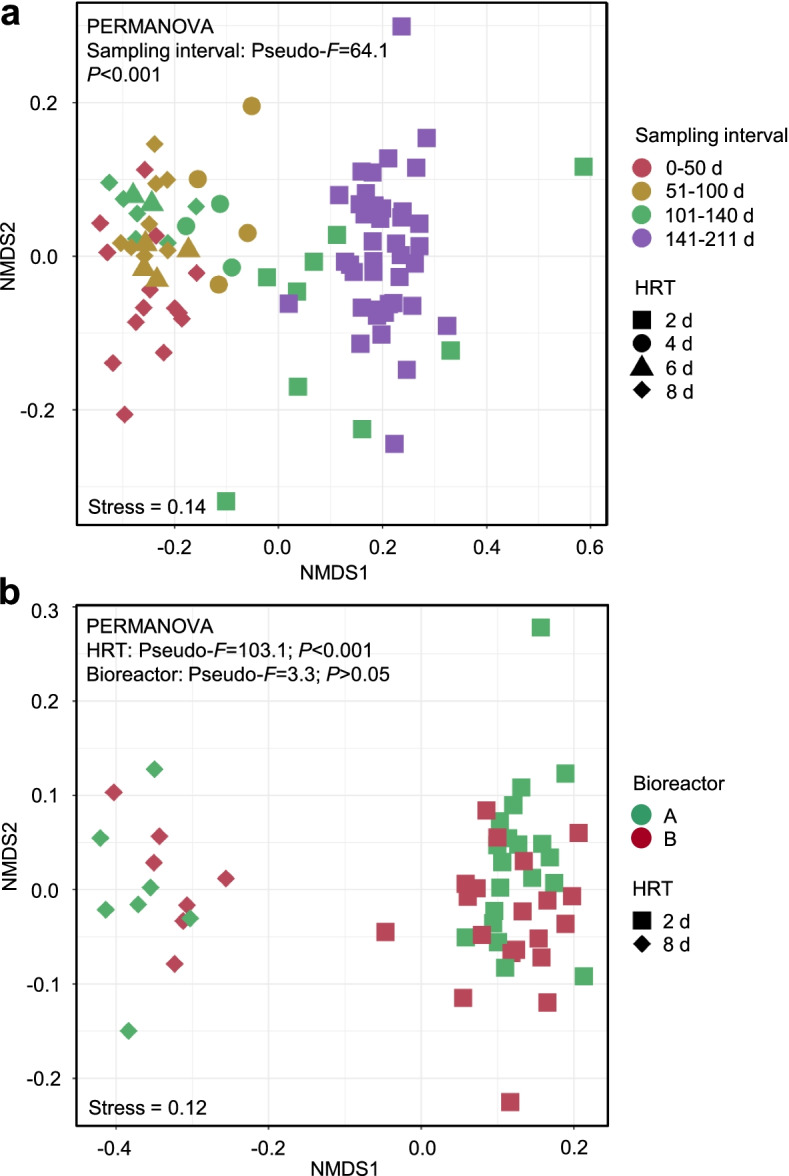
Dissimilarities in bacterial community composition (beta-diversity). Non-metric multidimensional scaling (NMDS) based on Bray-Curtis dissimilarities of microbial community composition in bioreactors. **a** All samples in the four HRT phases were considered for dissimilarity calculation. **b** Samples in the 8-day HRT phase classified to the sampling interval 0–50 days and in the 2-day HRT phase classified to the interval 141–211 days were included

The composition and diversity of the reactor microbiota varied when decreasing the HRT. Changes in the relative abundance of ASVs categorized from phylum to genus between the HRT of 8 days and 2 days are shown in Additional file 1 (Fig. S3). Alpha diversity metrics showed significantly lower observed ASV counts (pairwise *t*-test, *P* < 0.05) and higher Shannon index values (pairwise *t*-test, *P* < 0.05) for HRT of 8 days compared with 2 days (Additional file 1: Fig. S4). Beta diversity analysis revealed a significant difference between the communities at different HRTs (PERMANOVA; Pseudo-*F* = 103.1, *P* < 0.001) but no significant difference between the communities in both reactors at the same HRT (Pseudo-*F* = 3.3, *P* > 0.05) (Fig. 2).

### Selection of HRT bioindicators

**Fig. 3:**
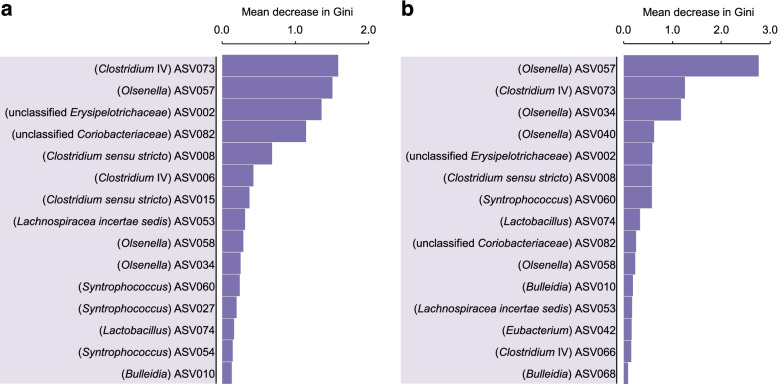
Random forest feature importance of ASVs used to classify the HRT phases (A-HRT bioindicators and B-HRT bioindicators). The top-ranked 15 ASVs reducing the uncertainty in the prediction of HRT phases (HRT of 8 days and 2 days). According to their ASV abundances distribution, the order of features (from top to bottom) was based on their mean decrease in Gini scores, with HRT as the response variable. **a** Feature importance of A-HRT bioindicators. The ASV importance was calculated using the relative abundance data of bioreactor A as a training set and data of bioreactor B as a test set. **b** Feature importance of B-HRT bioindicators. Similar to A-HRT bioindicators, ASV importance of B-HRT was calculated using the relative abundance data of bioreactor B as a training set and data of bioreactor A as a test set. The taxonomic classification of ASVs assigned at the genus level is provided in parentheses

To determine HRT bioindicators, we used HRT of 8 days and 2 days as prediction objects and relative abundances of ASVs as features. Different algorithms integrated into the mAML automated machine learning pipeline [26] were tested heuristically to choose the most fitting machine learning algorithm for our dataset. We selected random forest since it can rank feature relevance and it showed the highest prediction accuracies during the 5-fold cross-validation process (Additional file 2). We measured the prediction strength of our models in two folds. First, we trained the models using the data from bioreactor A and then tested them using bioreactor B. After we trained the models using the data from bioreactor B and tested them using bioreactor A. We selected the 15 top-ranked ASVs that gave the best discrimination between the HRT phases, based on higher than 1% of the mean decrease in Gini scores for both reactors in the prediction accuracy of HRT. The 15 most relevant ASVs to identify HRT changes were defined as “A- or B-HRT bioindicators”, potentially reflecting the key species correlating with HRT changes in either bioreactor (feature importance in Fig. 3). The two bioreactors shared 11 HRT bioindicators assigned to nine different genera.

### Prediction of process performance

To answer whether HRT bioindicators can be used to predict process performance in terms of C6 and C8 productivity, we performed a regression analysis. We created regression models using the dataset with the original distribution of samples, i.e., 14 samples from HRT 8 days and 40 samples from HRT 2 days, equally divided among the two different bioreactors. We also created regression models using artificially balanced datasets. We used the Synthetic Minority Oversampling Technique (SMOTE) to oversample the training datasets to have 100 samples with a balanced distribution of the two HRT classes. The datasets from bioreactors A and B were trained and tested independently. Consequently, we had the following experimental configuration: models were trained with the original dataset from bioreactor A/B and tested with the samples from bioreactor B/A; models were trained with the oversampled dataset from bioreactor A/B and tested with the samples from bioreactor B/A. Finally, all created models were evaluated with 5-fold cross-validation.

HRT bioindicators were first chosen as features to train the models. Considering that community assembly is affected by time, we then determined the 15 ASVs most relevant to each non-HRT process parameter (i.e., concentrations of lactate, C4, C6 and C8; productivities and yields of C4, C6 and C8; hereafter, non-HRT bioindicators). Initially, we trained regression models using three different machine learning algorithms: linear regression algorithm, support vector machine with radial kernel and random forest for regression. We used root mean squared errors (RMSE) as the evaluation metric, and the results are visualized as boxplots in Additional file 1 (Fig. S5 for the HRT bioindicators and Fig. S6 for the non-HRT bioindicators). The random forest regression algorithm performed overall better than linear regression and support vector machine with radial kernel. When using the HRT bioindicators as features for the regression, the random forest algorithm had the lowest RMSE median in 7 out of the 8 tested configurations, as shown in Additional file 1 (Fig. S5). In addition, the model trained with random forest showed consistency when comparing its performance in the original and the balanced datasets, which indicates that this algorithm is able to handle the imbalance present in our dataset. Therefore, the random forest for regression algorithm was selected as the best algorithm to determine HRT bioindicators. In our case, random forest could explain more than 80% of the variance in C6 and C8 productivities (Additional file 1: Tables S2-S3).

**Fig. 4:**
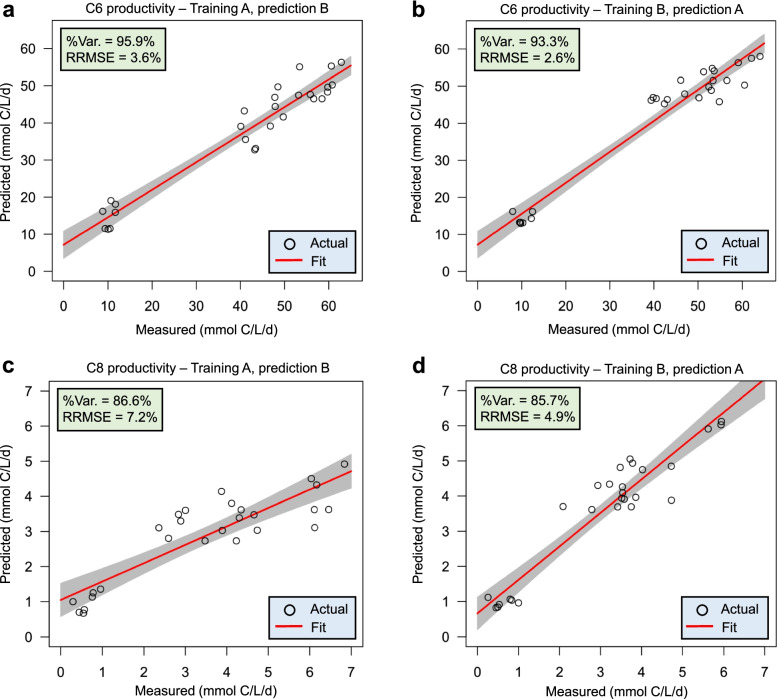
Prediction results of C6 and C8 productivities using HRT bioindicators. **a**, **b** Prediction performance of C6 productivity. **c**, **d** Prediction performance of C8 productivity. We obtained results in **a** and **c** by using relative abundance data of bioreactor A for training the model and data of bioreactor B for testing. Results using the data of bioreactor B for training and bioreactor A for testing are shown in **b** and **d**. The red lines and grey shaded areas depict the best-fit trendline and the 95% confidence interval of the least-squares regression, respectively. C6, *n*-caproate; C8, *n*-caprylate; %Var., explains the variance (%) in C6/C8 productivity of the training set; RRMSE, relative root mean square error

**Fig. 5:**
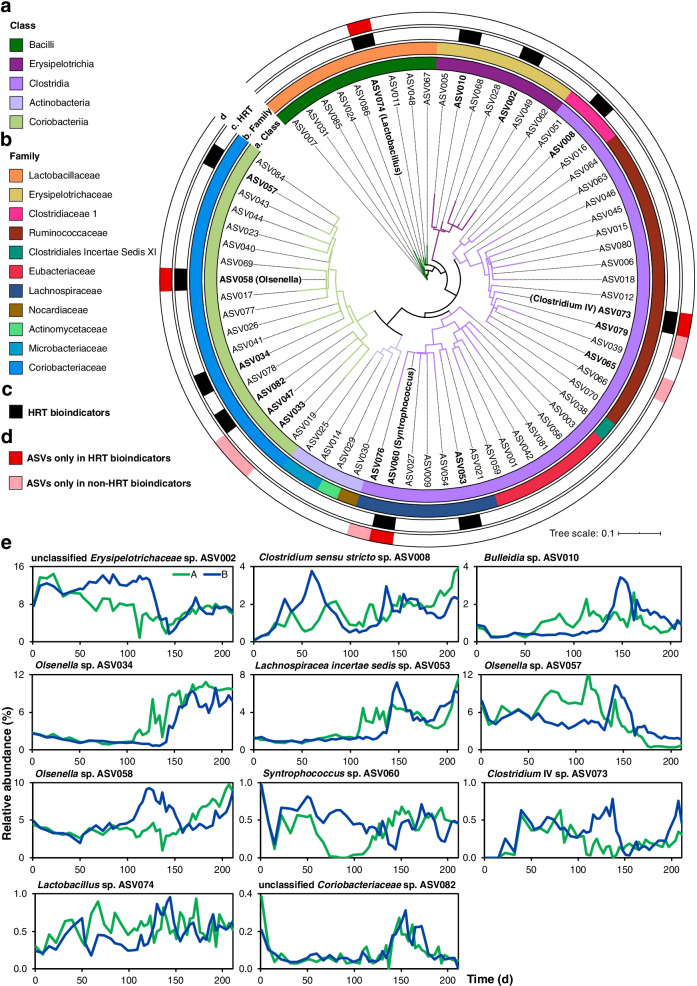
Phylogeny of HRT bioindicators and non-HRT bioindicators for considering community assembly caused by time. **a**, **b** A maximum likelihood 16S rRNA gene tree showing the ASV species based on the rarefied sequencing data. ASVs are coloured according to the class (**a**, first inner ring) and family (**b**, second inner ring). **c** The third inner ring shows the 11 HRT bioindicators identified in both reactors to predict HRT phases of 8 days and 2 days. The ASVs identified as HRT bioindicators are shown in bold. Their taxonomic assignments at the genus level are provided in the legend. **d** The four ASVs of HRT bioindicators irrespective of time are shown in red in the outer ring. The ASVs only present in non-HRT bioindicators of C6/C8 productivity are shown in pink in the outer ring. **e** Relative abundance dynamics of HRT bioindicators during the whole reactor operation period. In the legend, A and B stand for bioreactors A and B, respectively. The four ASVs (in bold) of HRT bioindicators, irrespective of time, assigned at the genus level are indicated in parentheses. C6, *n*-caproate; C8, *n*-caprylate

Using the selected random forest for the regression algorithm, we evaluated its prediction performance by comparing the process parameters' predicted and measured values. The average relative root mean square error (RRMSE) for the predictions made using the HRT bioindicators was 4.6% (Fig. 4), and the average RRMSE for the predictions made using the non-HRT bioindicators was 5.8% (Additional file 1: Fig. S7). We further tested samples in all HRT phases with HRT and non-HRT bioindicators. In all cases, the predicted C6 and C8 productivities showed RRMSE below 7.2% (Additional file 1: Figs. S8 and S9). Therefore, we considered HRT bioindicators irrespective of time as the ASVs presented in HRT bioindicators and not in non-HRT bioindicators (feature importance in Additional file 1: Figs. S10 and S11). Interestingly, the same four ASVs assigned to the genera *Olsenella*, *Lactobacillus*, *Syntrophococcus* and *Clostridium* IV were identified for C6 and C8 productivity (Fig. 5). We thus hypothesize that species represented by these four ASVs determined the increased C6/C8 productivities in the CE process manipulated by changing operational conditions, i.e., shortening the HRT.

### Functional role of HRT bioindicators

**Fig. 6:**
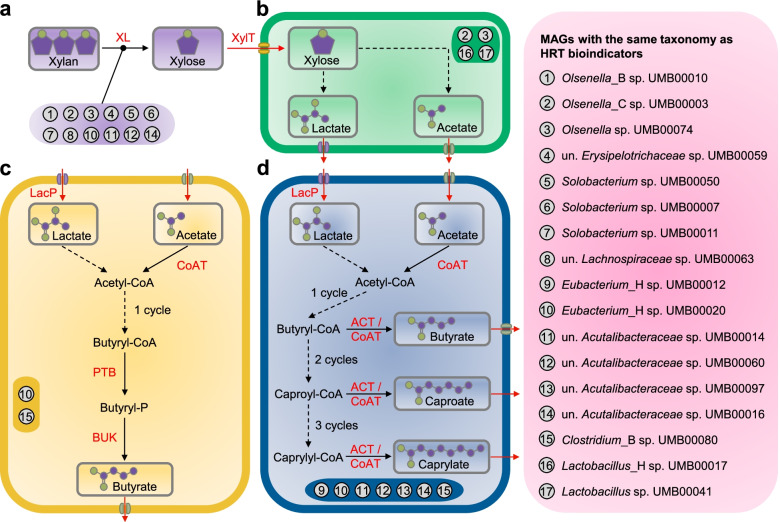
Genetic potential of metagenome-assembled genomes (MAGs) with the same taxonomy as HRT bioindicators driving the catabolism of xylan and lactate to *n*-caproate and *n*-caprylate. These catabolic steps were categorised into four main functions of the anaerobic mixed culture fermentation. **a** Hydrolysis of xylan. **b** Xylose fermentation producing acetate and lactate. **c** Butyrate formation from lactate and acetate. **d** Chain elongation with lactate as electron donor producing *n*-butyrate, *n*-caproate and *n*-caprylate. Numbers represent the 18 different MAGs with similar phylogenies as the HRT bioindicators at the genus level (details in Table 1). The enzyme abbreviations are provided in red letters next to the pathways (solid lines). Dashed lines represent multi-enzyme reactions between the two indicated molecules. In (**d**), “cycle” refers to the reverse β-oxidation cycle. The complete metabolic pathways are depicted in Additional file 1: Fig. S12. un., unclassified; XL, xylanase (EC 3.2.1.8); XylT, xylose transporter (EC 7.5.2.10, EC 7.5.2.13); LacP, lactate permease (TC 2.A.14); CoAT, butyryl-CoA:acetate CoA-transferase (EC 2.8.3.-); PTB, phosphate butyryltransferase (EC 2.3.1.19); BUK, butyrate kinase (EC 2.7.2.7); ACT, acyl-CoA thioesterase (EC 3.1.2.20)

**Fig. 7:**
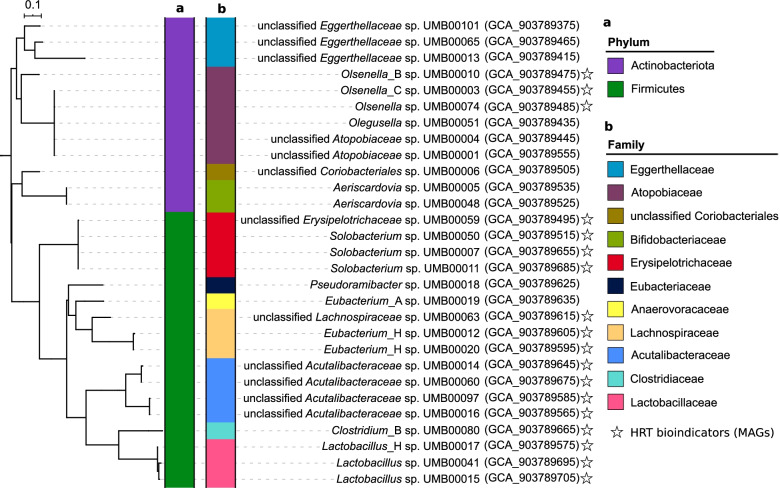
Phylogenetic tree of the recovered metagenome-assembled genomes (MAGs). **a**, **b** A phylogenomic tree based on mash distances showing the MAGs taxonomy determined by GTDB-Tk at phylum (**a**) and family (**b**) levels. A total of 108 MAGs were recovered and differentiated into 29 species based on the ANI values. We defined the representative MAG for each species as that showing high quality. Only the representative MAG for each species is depicted in the tree. The ENA accession numbers of the representative MAGs are shown in parentheses. MAGs with similar phylogenies as HRT bioindicators are indicated by a star

Genomic information on the species of HRT bioindicators indicated their roles in driving the catabolism of xylan and lactate to C6/C8 (Fig. 6). Details of the whole genome sequencing can be found in the section “Metagenomic analysis” (“Methods” section). Among 108 metagenome-assembled genomes (MAGs; dereplicated into 29 species; Fig. 7 and Additional file 3), we recovered 12 species with similar phylogenies as the four genera representing the HRT bioindicators (Table 1). In view of the fermentation process, we annotated the genetic potential for xylan hydrolysis, xylose fermentation and CE with lactate (Additional file 1: Fig. S12 and Additional files 4, 5, 6 and 7). Specifically, *Clostridium* IV species were reported as lactate-based chain-elongating bacteria [28]. Our results suggest that four *Clostridium* IV species (*Acutalibacteraceae* spp. according to GTDB-Tk) can convert lactate to C6/C8. Two *Syntrophococcus* species (*Eubacterium*_H spp. according to EZBioCloud [29]) are potential C6/C8-producers as they hold complete gene sets encoding enzyme complexes that catalyze CE reactions. This genetic potential was also found in genomes of closely related *Syntrophococcus* species (*Eubacterium cellulosolvens* according to EZBioCloud; Additional file 7), which was not described before. Lactate formation from xylose by lactic acid bacteria can enhance CE by providing additional electron donors [30–34]. A recent study reported an enriched community dominated by *Lactobacillus* and chain-elongating species, and their co-occurrence suggested lactate produced by *Lactobacillus* to be a key intermediate for C6/C8 production [35]. Network analysis of our previous study [10] revealed the co-occurrence of *Olsenella* with potential chain-elongating species. Species of *Lactobacillus* and *Olsenella* are potential xylose-consuming lactate producers (Fig. 6b). Genes encoding xylanases were not found in *Lactobacillus* MAGs but in those assigned to other bioindicators (Fig. 6a). Taken together, the delineated synergy effects between these bioindicator species suggest a division of labor with mutual benefits, converting xylan and lactate to C6/C8. A correlation network shows HRT, C6 and C8 productivity being the most highly connected nodes (Additional file 1: Fig. S13). Their co-occurrence with ASVs assigned to *Clostridium* IV, *Olsenella* and *Syntrophococcus* indicates strong associations among these taxa, the changing environment and corresponding functions. The predictability of C6 and C8 productivities was relatively poor when using only the four HRT bioindicators irrespective of time (Additional file 1: Fig. S14). Besides, we found redundancy in the main functions of catabolizing xylan and lactate to C4, C6 and C8 (Fig. 6), with the relevant HRT bioindicators increasing in relative abundances (Additional file 1: Fig. S15). Thus, the involved metabolic pathways seem to be strongly coupled to HRT decrease. The genetic potential overlaps with that of other distinct taxa of the reactor microbiota, suggesting that HRT bioindicators might be key species of the process, but ecological interactions with other species are critical to ensure the C6/C8 production (functional annotations of xylose fermentation and chain elongation in Additional files 6 and 7).

**Table 1:**
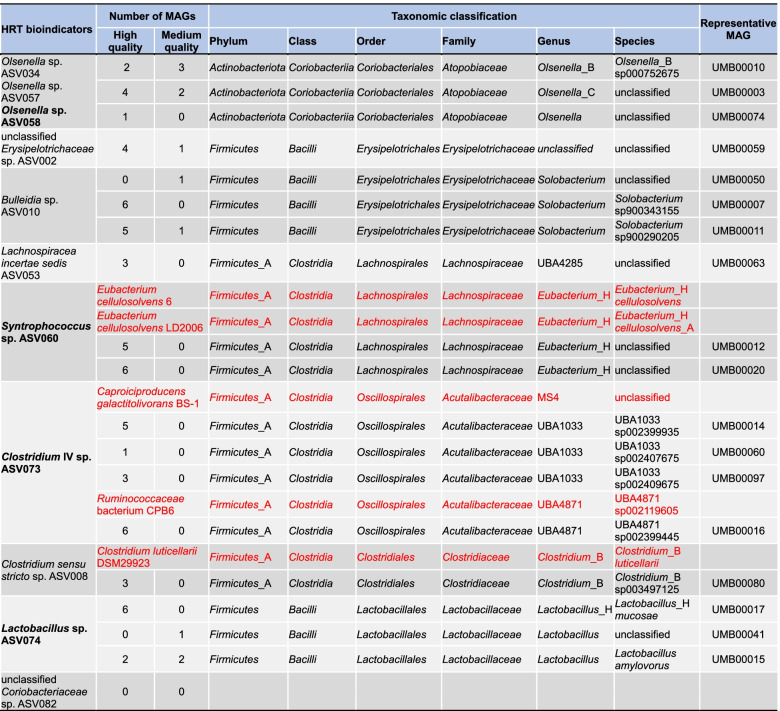
Summary of metagenome-assembled genomes (MAGs) with the same taxonomy as HRT bioindicators

Taxonomy refers to the GTDB (Genome Taxonomy Database) phylogenomic classification. ASVs in bold represent the four HRT bioindicators irrespective of time. Sequence datasets of genomes in red letters were taken from the databases of NCBI and EzBioCloud. These genomes (in red) were used to affiliate the MAGs of *Syntrophococcus*, *Clostridium* IV and *Clostridium sensu stricto*, since their genomes are not available in GTDB. See details of MAGs in Additional file 3: Dataset S1. *ASV* amplicon sequencing variant

## Discussion

### Bioreactor performance and community dynamics

Continuous reactor systems maintain cultures in a specific growth rate and physiological state [36]. Therefore, these systems are perfect for the exploration of CE as a biotechnological platform for continuous production of medium-chain carboxylates [9]. In this study, we used continuous anaerobic bioreactors with the enriched chain-elongating microbiota [10] as model ecosystems. Two reactors were operated in parallel starting from one inoculum, thus representing biological replicates, and with frequent sampling over 211 days. We demonstrated that shortening the HRT from 8 to 2 days improved C6/C8 productivity and caused specific shifts in the microbial community in both reactors independently of the temporal scheme applied for HRT reduction (i.e., gradual decrease vs. fast transition mode). As we had stable biomass concentrations and detected certain species at all times, we can make sure that these species were growing in each bioreactor since otherwise they would have been washed out from the reactor microbiome. Using multivariate analysis, we demonstrated that the microbial communities established at 8 days HRT were different from those at 2 days HRT. These analyses also showed that the microbial communities sampled from the two reactors at the corresponding HRT regime were not significantly different (PERMANOVA, *P* < 0.05). Commonly only two lab-scale reactors are run in parallel for long-term experiments with complex reactor microbiomes [35, 37–40]. In contrast to natural ecosystems with their spatial and temporal heterogeneities and uncontrollable environmental factors, bioreactors represent highly controlled model ecosystems that can be sampled at high frequency over long experimental periods, thereby accounting for stochastic effects despite the comparably low number of biological replicates. The obtained time series data are robust and have been used, for instance, to explore pH effects on the CE process [41] and to unravel long-term successional patterns of community assembly in anaerobic processes [42].

### Evaluation of the machine learning approach

Machine learning methods can simultaneously incorporate the relative abundances of multiple ASVs and their context-dependency, surpassing traditional statistical approaches that consider each ASV in isolation (e.g., the empirical Bayes moderated t-statistics) [43]. Multivariate analysis has been shown to enable superior performance compared to individual analysis in the context of sensitivity, specificity and robustness, as it considers potential synergies between the features [44]. Therefore, we used a machine learning approach based on the retrieved 16S rRNA ASVs in two steps of the study: to identify potential bioindicators of HRT and to create predictive models of *n*-caproate and *n*-caprylate productivities.

To identify potential bioindicators, it is necessary to assess the value of the features from the microbiome in an unbiased way—identifying not only their statistical significance but also their prediction accuracy on independent samples [45]. Consequently, to increase the generality of our approach and to reduce any potential bias present in the samples, we systematically used samples from one bioreactor for training the machine learning models while using the samples from the other bioreactor for testing the model. On the other hand, deploying a machine learning solution is not trivial. To avoid over-optimistic results, it is important to consider the distribution and format of the training data and the intrinsic differences of the algorithms themselves [46].

When searching for the optimal manner of dealing with our data, we faced two potential problems: our dataset class distribution is imbalanced concerning the HRT classes (40 samples from 2 days HRT and 14 samples from 8 days HRT), and the total number of samples we have, which is 54, may be limiting to train a robust model. Most machine learning algorithms evaluate themselves during the learning process by comparing the predicted target with the original labeled sample. This creates a bias in the algorithms towards the majority target [47]. In addition, training models with small datasets may create overfitted models that are overly sensitive to outliers and noise. In this work, we first tested the separability of the two classes (HRT of 2 days and HRT of 8 days) by empirically testing several machine learning algorithms to differentiate those two classes using the samples’ ASV composition. As shown in Additional file 2, most algorithms were able to differentiate our targets. This analysis indicates that the features (ASVs) can potentially describe the complexity of our problem by characterizing the different communities at the two HRTs. We also tackled the imbalance and limited samples in our data by pre-processing our dataset to generate new samples using SMOTE. To evaluate the generality of our model, we systematically used samples from one bioreactor for training the machine learning models while using the samples from the other bioreactor for testing.

Finally, we also integrated a validation strategy into our machine learning pipeline. Validation is one of the most important techniques when creating a generalized model since it estimates the stability of the model when dealing with new data. The validation approach we used is the *k*-fold cross-validation. The general idea of using *k*-fold cross-validation was to train our model with a selected group of samples from our data and validate it with the remaining samples, rotate the training and validation groups *k* times until we used all samples to train a model, and all samples to validate a trained model. This approach provides much more confidence in the results by letting us use all the data to train different models [48].

Initially, we wanted to determine potential HRT bioindicators. Therefore, the initial step of our machine learning pipeline was to heuristically try several different classification algorithms to determine which of them can better differentiate 2 days HRT and 8 days HRT. To do so, we used the mAML pipeline to create classification models using several tree-based and non-tree-based classifiers systematically. Most of the algorithms had more than 90% classification accuracy. This indicates that the microbiome composition of 2 days HRT and 8 days HRT should be considerably different, and thus directly divisible. To select an algorithm, however, we also considered the ability of the algorithm to rank feature relevance, since we wanted to select the most important ASVs to differentiate the target HRT. Random forest has been shown to run efficiently and accurately on high-dimensional datasets with multi-features by constructing an ensemble of decision trees [49]. Further, it avoids overfitting by integrating out-of-bag estimates [49]. Finally, other studies that used 16S rRNA sequencing data in machine learning solutions also reported random forest to show good prediction performance [43, 50, 51]. For these reasons, we selected the random forest algorithm to extract HRT bioindicators.

Once we selected the potential HRT bioindicators, we developed regression models to predict *n*-caproate and *n*-caprylate productivities. Our machine learning solution for creating the regression models attempts to consider all the potential problems mentioned (i.e., selecting an adequate algorithm, dealing with an imbalanced dataset and potentially insufficient number of samples, avoiding overfitting and increasing the generality of the model). We evaluated three different regression algorithms with different biases: linear regression, support vector machine and random forest regression algorithm. In all cases, we balanced our dataset and increased the number of samples using the SMOTE. Boxplots were created to interpret the results of the 5-fold cross-validation visually.

Subsequently, we compared the results from the models created with the original and balanced datasets. Oversampling techniques of any kind can introduce bias to the data and create overfitted models. SMOTE tries to reduce oversampling bias by generating similar but not equal samples. Ideally, collecting more real samples should solve this problem in future studies. For instance, in Fig. S5, one can see that linear model regression caused a drastic reduction in RMSE when comparing S5e (trained with imbalanced dataset) and S5f (trained with balanced dataset). This indicates that linear model regression could not intrinsically deal with the imbalance in our dataset, and the model created using the balanced dataset may have been overfitted. Although not as drastic as the linear model, the other two tested regression algorithms (SVM with radial kernel and random forest) also reduced their RMSE when trained with the SMOTE datasets. This reduction could be attributed to the balancing method that did not introduce much variance to the dataset since the new samples are slightly different from the original ones. However, random forest showed consistently good predictive performance. Hence, this may indicate that random forest for regression can naturally deal better with our imbalanced dataset. Consequently, we decided to use random forest for regression and our original data samples distribution to create our final prediction models. It is also relevant to mention that we trained the prediction models with samples from one bioreactor and tested with the other, thereby reducing the risk of overfitting.

However, random forest is not the only machine learning algorithm used for predictive analytics in microbiome studies. For example, with an integration of the phylogenetic tree information into the predictive framework, the recently proposed phylogeny-regularised sparse generalized linear model [52] and regression model [53] showed superior prediction power in real microbiome dataset applications. Using human gut microbiome data for continuous age prediction, the so-called glmmTree model achieved the best performance as indicated by the highest *R*^2^ of 70% and the lowest predicted mean square error of a median value 1.3, with a 5-fold cross-validation being applied [52]. The random forest algorithm used in this study achieved results comparable to the glmmTree model with *R*^2^ over 80%.

### Function of bioindicator species in chain elongation

Mining the functional potential of MAGs affiliated to bioindicators may indicate key functions of these species in the CE process. In particular, the MAGs of the lactate-based CE species such as *Clostridium* IV revealed all genes necessary for lactate oxidation and CE by reverse β-oxidation. To validate this hypothesis, we also annotated the genome of the chain-elongating *Ruminococcaceae* bacterium CPB6 affiliated to *Clostridium* IV [28], which contains complete gene sets encoding enzyme complexes for converting lactate to C6. Interestingly, our results revealed novel species with the genetic potential for chain elongation. Our results may guide other researchers studying CE to characterize novel chain-elongating bacteria in previously reported CE microbiomes.

Here, we used metagenomics to unravel the function of key species in CE that were inferred from 16S rRNA sequencing data. Details of the whole genome sequencing can be found in the section “Metagenomic analysis” (“Methods” section). This functional analysis is more reasonable than inferring the function of species based on the 16S rRNA sequencing data, but the genetic potential alone does not guarantee that the respective metabolic process is performed [54]. Therefore, follow-up studies involving multi-omics are necessary to verify if the genetic potential found in the MAGs corresponds with active pathways. Besides multi-omics experiments, the novel genetic information related to the CE process could be validated in wet-lab experiments using defined mixed cultures of isolated strains representing the bioindicator species [55]. By constructing synthetic microbial consortia with different combinations of those representative bioindicator species and monitoring their growth and metabolic behavior under controlled conditions, mechanistic and metabolic modeling could be used to verify the ability of our machine learning framework to predict ecophysiological functions from 16S rRNA sequencing data.

### Engineering microbial communities for bioprocesses with distributed pathways

**Fig. 8:**
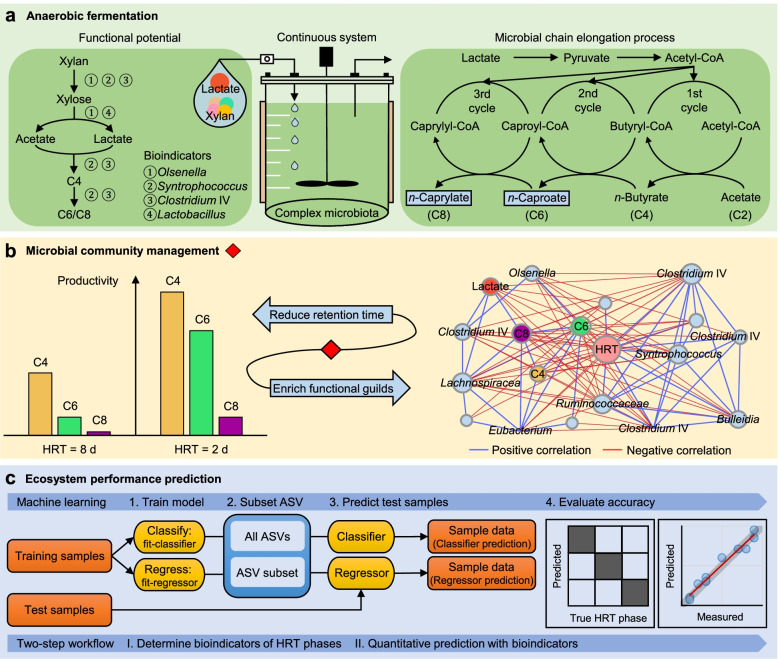
Overview of the quantitative prediction of process performance in the anaerobic bioreactor system. **a** Anaerobic mixed culture fermentation of lactate and xylan for the production of *n*-caproate (C6) and *n*-caprylate (C8) by lactate-based chain elongation. Based on the recovery of metagenome-assembled genomes, the left panel shows the bioindicators capable of performing key steps of the fermentation. **b** Reducing the hydraulic retention time (HRT) as an operation-based strategy to optimise the process performance and to manage the reactor microbiota towards desired functions. Shortening the HRT from 8 to 2 days enhanced productivities of C4, C6 and C8. The enriched reactor microbiota comprised functional groups involved in xylan hydrolysis, xylose fermentation and chain elongation with lactate, presented by a co-occurrence network of environmental factors (controlled conditions with only reducing the HRT), ecosystem functioning (process performance) and microbial community. The full network is shown in Additional file 1: Fig. S13. **c** Predicting performance of ecosystem processes with random forest analysis. We developed a random forest two-step workflow to predict the HRT phases and carboxylate production quantitatively by using relative abundance data of the 16S rRNA-derived species (ASVs, amplicon sequence variants)

In engineered and natural ecosystems, phylogenetic diversity can be linked to ecosystem processes in which microbial communities perform key functions [56]. The machine learning approach used in the current study enabled the quantitative prediction of community functioning (i.e., CE) in the anaerobic bioreactor system (Fig. 8). Converting xylan and lactate to medium-chain carboxylates is a complex metabolic process consisting of mainly four functions; i.e., xylan hydrolysis, xylose fermentation, C4 formation from lactate and acetate and CE with lactate producing C4, C6 and C8, with more than 30 enzymes being involved. We showed that alternative pathways can be used for this complex conversion (Additional file 1: Fig. S12). Because of this complexity, it is likely that the observed increase in C6/C8 productivity after shortening HRT from 8 to 2 days was not driven by a single microorganism but by the joint effort of multiple species within our bioreactors. However, not all species in the bioreactor were directly involved in CE. Our feature selection approach helped us identify the species linked to metabolic pathways potentially involved in CE. This was possible because we included quantitative metadata such as time-series data of substrate and product concentrations, which facilitated to filter species linked to the CE process. A similar analysis identified key species that could predict the overall quality of soils [25]. In the latter study, the authors showed that using the soil bacterial community indicators associated with metadata of soil physicochemical variables facilitated to predict the soil quality with 50–95% accuracy [25].

We also provided new biological insights into the reactor microbiomes of lactate-based CE. The importance of in situ lactate formation in the lactate-based CE process has been emphasized by several studies [30–35]. Our results indicate that species of the genera *Lactobacillus* and *Olsenella* are potential xylose degraders but *Lactobacillus* species cannot utilize the polysaccharide xylan due to the lack of genes encoding xylanases. This result indicates different functional roles of lactic acid bacteria in the degradation of biomass residues containing hemicellulose, which was reported to be more degradable than cellulose during acidogenic fermentation of maize silage [57]. These new insights into the microbial ecology of the CE process may open doors for further valorisation of carbohydrate-rich waste streams. For example, bioaugmentation of xylan-hydrolysing lactic acid bacteria such as *Olsenella* species in CE communities may optimize the breakdown of hemicellulosic compounds. In addition, we demonstrated that C4 is not only produced by CE of acetate but also from xylose by butyric acid fermentation [27], which competes with CE in the recovery of carbon from sugars. This xylose fermentation to C4 was also described as a competing process in other CE studies [10, 58]. Currently, it is still a challenge to steer the CE community functioning to only medium-chain carboxylates in the mixed culture fermentation, but the direction of creating synthetic microbial consortia with modularity (e.g., spatial niches) could be a wise option to mediate a multi-step bioprocess and to utilize metabolic diversity in any single reactor system [59].

In our engineered ecosystems with well-controlled conditions (temperature, pH and no immigration of other microbes; Fig. 8a), HRT was the most influencing factor controlling community assembly (Fig. 8b). However, we cannot exclude the impact of other deterministic factors like microbial interactions within temporal patterns, particularly for such a long-term reactor experiment. When the random forest regression models took time instead of HRT into account, the results indicated that the non-HRT bioindicators might result from the intrinsic community dynamics alone. Thus, prediction results of the HRT bioindicators can be biased by these autoregressive data present in time series. Even though the HRT bioindicators irrespective of time seem to be key species for the increase in C6/C8 productivity caused by HRT decrease, we cannot ignore the contribution of the non-HRT bioindicators to community assembly and functioning, particularly with functional redundancy shown in the main functions of the CE process. Therefore, the effects of compositional stochasticity on community assembly also need to be considered [60, 61]. Further studies on these ecological principles will help manage reactor microbiota towards beneficial traits, such as high specificities for C6/C8 production.

## Conclusions

The continuous reactor systems with enriched communities facilitated the selection of reactor microbiomes with desired CE functions (i.e., high C6 and C8 productivities). We demonstrated that 16S rRNA amplicon sequencing data could be used to predict CE process performance quantitatively (> 90% accuracy). The described machine learning framework (Fig. 8c) may be suitable for other ecosystem processes and more complex communities. For that, it would be necessary to design experiments with (i) sufficient temporal and/or spatial resolution, (ii) parallel sampling for amplicon sequencing data and metadata from desired ecosystem processes and (iii) correlation of phylogenetic diversity with the ecosystem processes. Our approach was based on phylogenetic diversity (relative ASV abundances) that, in some ecosystems, may correlate with ecosystem processes where microbiota perform key functions. Due to the use of unbalanced datasets, the high dimensionality and more direct link with different ecosystem processes found in omics data, our general methodology can be adapted to other data types, including functional genes, transcripts, proteins or metabolites. Our approach opens new doors for prediction and hypothesis testing in microbiome research. Further studies are needed to reveal which data types reflect different ecosystem processes and communities with different levels of complexity.

## Methods

### Reactor operation and monitoring of process parameters and community composition

The inoculum was initially taken from a continuous lab-scale bioreactor that produced C6 and C8 by anaerobic fermentation of lactate-rich corn silage [11]. Enrichment was performed in a reactor that was daily fed with mineral medium (pH 5.5; Additional file 1: Table S4) containing water-soluble xylan (more than 95% xylooligosaccharides, from corncob; Roth, Karlsruhe, Germany) and lactic acid (85%, FCC grade; Sigma Aldrich, St. Louis, USA) as defined carbon sources and produced C4, C6 and C8 over 150 days [10]. For the present study, two 1-L bioreactors (A and B; BIOSTAT® A plus, Sartorius AG, Göttingen, Germany) were filled up with 0.5 L of the enriched culture. Both bioreactors were daily fed with 0.125 L medium containing 1.47 g lactic acid and 1.25 g xylan, without withdrawing effluent. After 4 days, the contents of both bioreactors were mixed by pumping them three times from bioreactor A to B and back while keeping anoxic conditions. Eventually, they were equally distributed to both bioreactors, which is considered the starting point (day 0) of the experiment.

We employed semi-continuous stirred tank reactors for anaerobic fermentation, which were operated at 38 ± 1 °C and constantly stirred at 150 rpm. The pH of the reactor broth was automatically controlled at 5.5 by addition of 1 M NaOH. For each bioreactor, the produced gas was collected in a coated aluminium foil bag that also served for compensating underpressure in the reactor system. The bag was connected after a MilliGascounter® (MGC-1; Ritter, Bochum, Germany) that measured on-line the volume of the produced gas. A gas-sample septum was placed in the gas pipe of each bioreactor.

In the beginning, both bioreactors were operated as duplicates with an equal HRT of 8 days. For daily feeding, 1.47 g lactic acid and 1.25 g xylan were supplied in mineral medium. After 51 days, we gradually decreased the HRT of bioreactor A from 8 to 6 days, and further to 4 days and 2 days while the operation of reactor B was continued at HRT of 8 days as a control as shown in Additional file 1: Table S5. Next, we shortened the HRT of bioreactor B from 8 to 2 days in a fast transition mode and with the same substrate load as in bioreactor A, in order to reproduce the HRT transition in the second reactor. Considering the effect of time on community assembly, we conducted unequal HRT changes in the two bioreactors and aimed to delineate the model prediction strength with the two different datasets. Finally, both bioreactors were operated in parallel at an HRT of 2 days until day 211.

Gas samples were taken through the septum twice per week. Samples for measuring optical density (OD) and for DNA extraction were collected twice per week from the reactor effluent. Concentrations of xylan, carboxylates and alcohols were measured in the effluent supernatants [10]. In total, effluent samples were collected on 59 time points for each bioreactor. At the beginning and the end of the experiment, pelleted biomass from the effluent was used to determine the cell dry mass as previously described [10]. For microbial community analysis, pelleted cells from 2 mL effluent were washed with 100 mM Tris-HCl pH 8.5 and stored at − 20 °C until DNA extraction.

### Analytical methods

Daily produced gas volume was monitored with the MGC-1 and normalized to standard pressure and temperature [30]. Gas composition (H_2_, CO_2_, N_2_, O_2_ and CH_4_) was determined by gas chromatography in triplicate [62]. Concentrations of carboxylates and alcohols were analyzed in triplicate by gas chromatography [10]. The concentration of xylan was measured by a modified dinitrosalicylic acid reagent method [10]. Cell mass concentration was calculated from OD values that were correlated with the cell dry mass [10]. The calculated mean correlation coefficients were 1 OD_600_ = 0.548 g L^−1^ for bioreactor A and 1 OD_600_ = 0.537 g L^−1^ for bioreactor B.

### Microbial community analysis

Total DNA was isolated from frozen cell pellets sampled twice per week using the NucleoSpin® Microbial DNA Kit (Macherey-Nagel, Düren, Germany). Methods for DNA quantification and quality control were as described previously [63]. For high-throughput amplicon sequencing, V3–V4 regions of the 16S rRNA genes were PCR-amplified using primers 341f and 785r [64]. Sequencing was performed on the Illumina Miseq platform (Miseq Reagent Kit v3; 2 × 300 bp). A total of 12,168,404 sequences ranging from 57,612 to 389,963 pairs of reads per sample (mean: 135,205; median: 122,367) were obtained.

The demultiplexed sequence data were processed with the QIIME 2 v2019.7 pipeline [65] using the DADA2 plugin [66]. The DADA2 parameters were set as follows: trim-left-f 0, trim-left-r 0, trunc-len-f 270, trunc-len-r 230, max-ee 2 and chimera-method consensus. A total of 4,194,700 sequences ranging from 13,518 to 138,498 reads per sample were retained, with a mean of 46,608 reads per sample. The generated feature table indicates the frequency of each ASV clustered at 100% identity. Taxonomic assignment was done with a naïve Bayes classifier trained on 16S rRNA gene sequences of the database MiDAS 2.1 [67] and curated using the RDP Classifier 2.2 with a confidence threshold of 80% [68]. For downstream analyses, ASVs of all samples were rarefied to a sequencing depth of 13,518 reads (rarefaction curve reached the plateau, Additional file 1: Fig. S16). We obtained a total of 71 unique ASVs in 90 samples (ASV table and taxonomy table in Additional file 8).

Alpha diversity based on rarefied ASV data was evaluated by the observed ASV counts and the Shannon index [69], which were determined using the R package phyloseq v1.30.0 [70]. Dissimilarities in bacterial community composition (beta-diversity) were calculated using Bray-Curtis distance [71] based on rarefied ASV abundances and visualized as nonmetric multidimensional scaling (NMDS) plots. Statistical analyses of beta-diversity results were performed using permutational multivariate analysis of variance (PERMANOVA) [72] in the R package “vegan” (v2.5.6, “adonis” function, Monto-Carlo test with 1000 permutations); *P* values were adjusted for multiple comparisons using the false discovery rate (FDR) method [73].

### Network analysis

The co-occurrence network analysis was performed using the method described by Ju et al. [74]. Briefly, we constructed a correlation matrix by computing possible pairwise Spearman's rank correlations using the rarefied ASV abundances and abiotic parameters (HRT; concentrations of C4, C6, C8 and lactate; productivities and yields of C4, C6 and C8). Correlation coefficients below − 0.7 or above 0.7 and adjusted *P*-values (FDR method) lower than 0.05 were considered statistically robust. Network visualization and topological feature analysis were conducted in Gephi (v0.9.2) [75].

### 16S rRNA phylogenetic analysis

The 16S rRNA gene sequences of ASVs were aligned using the SINA alignment algorithm [76] via the SILVA web interface [77]. We additionally used SINA to search and classify the sequences with the least common ancestor method based on the SILVA taxonomy. For each query sequence, the minimum identity was set to 0.95 and the five nearest neighbors were considered. The tree was reconstructed based on the aligned sequences and their neighbors, with RAxML using the GTRCAT model of evolution. Later only ASV species of this study were kept in the generated tree for easier viewing. The tree was visualized using iTOL [78].

### Metagenomic analysis

Six samples from the previous enrichment experiment [10] were selected for whole-genome sequencing, which was performed by StarSEQ GmbH (Mainz, Germany) using the Illumina NextSeq 500 system (NEBNext Ultra II FS DNA library prep kit; 2 × 150 bp) with at a minimum of 20 million reads per library generated. Quality check and reads trimming were performed using metaWRAP (v0.7, raw read QC module) [79] and TrimGalore (v0.4.3) [80]. Reads of human origin were discriminated from microbial reads using BMTagger (v3.101) [81]. All adapters were removed and the resulting reads were assembled using metaSPAdes (v3.11.1) [82]. Paired-end reads were aligned back to the assembly using BWA (v0.7.15, mem algorithm) [83]. Binning of assembled contigs was performed using the metaWRAP modules metaBAT (2.12.1) [84], MaxBin (2.2.4) [85] and CONCOCT (1.0.0) [86]. The metaWRAP-Bin_refinement module was applied to separate the overlaps between two bins. Quality of MAGs was checked using CheckM (v1.0.7) [87]. MAGs were classified in high or medium quality regarding completeness, contamination, quality score (completeness − 5 × contamination) and strain heterogeneity [88]. The following thresholds were used for high quality: quality score > 50, completeness > 80, contamination < 5 and strain heterogeneity < 50; and for medium quality: quality score > 50, completeness > 50 and contamination < 10. One bin with lower quality was removed from the analysis. The taxonomy was assigned using GTDB-Tk (v0.3.2) [89]. Genome metrics were calculated with the statswrapper tool in the BBTools suite [90]. A phylogenomic tree based on Mash distances was generated with Mashtree (V1.1.2) [91] and visualized in iTOL [78]. Miscellaneous visualizations of the dataset metrics were performed in R with the packages ggplot2 (v3.3.0) and DataExplorer (v0.8.1). Species differentiation was performed using fastANI [92] and aniSplitter.R (http://github.com/felipborim789/aniSplitter/). Genomes were annotated with Prokka (v1.14.6) [93]. Functional annotation of genes relevant to xylan hydrolysis, xylose fermentation and chain elongation was curated using Swiss-Prot, COG and GenBank [94–96]. Default settings were chosen for all tools unless otherwise specified.

### Determination of bioindicators of HRT changes

To select the machine learning algorithm for differentiating the HRT phases of 8 days and 2 days, the mAML automated machine learning pipeline [26] was used to test several different algorithms on our microbiome data heuristically. We selected the algorithm with the highest prediction accuracy to rank feature relevance. ASV relative abundances were used as features to train and test the different classifiers included in the mAML pipeline. After the initial algorithm selection process, the random forest algorithm (randomForest R package, v4.6-14) [97] was chosen to determine the HRT bioindicators due to its high accuracy and ability to rank feature relevance. Considering how we replicated the HRT changing mode in both bioreactors (Additional file 1: Table S5), the whole operation period was divided into four sampling intervals: 0–50 days, 51–100 days, 101–140 days and 141–211 days. Based on the results of community analysis, we chose the ASV data of both bioreactors in the sampling intervals of 0–50 days and 141–211 days to determine the HRT bioindicators, and we used data of all samples in the four HRT phases as controls. To delineate the model prediction strength, we trained the classifier with ASV data of one bioreactor and tested in the other bioreactor and vice versa. For random forest classification analysis, importance of the different features (ASVs) was measured by the Gini index (mean decrease in Gini, default in randomForest R package, where larger values indicate a variable to be more important for accurate classification [98]).

The random forest classifier was trained on the training set, with 2000 trees and 40 variables (with the lowest out-of-bag estimated error rates achieved) being selected randomly for each tree. Explained variance (% Var. explained in R) was used to measure the model performance on the training set [97]. We predicted the accuracy by measuring how well the features can classify the HRT phases on the test set [98]. We first computed the feature importance of all 71 ASVs. Then in each step, the ASVs having the smallest importance were eliminated and a new forest was built with the remaining ASVs. For both bioreactors, the features were selected when their Gini scores were higher than 1% of the sum of the Gini scores of all ASVs (Additional file 9). Feature selection based on the random forest classifier with its associated Gini index has shown abilities to identify optimal feature subsets in high-dimensional data [99]. Finally, we selected the 15 top-ranked ASVs leading to the model of lowest error rate for classifying the HRT phases of 8 days and 2 days. In each bioreactor, the 15 ASVs that best discriminated between HRT phases were referred to as A-HRT bioindicators or B-HRT bioindicators (bioreactors A and B, respectively). ASVs common to both sets were defined as HRT bioindicators (workflow of random forest classification in Additional file 1: Fig. S17).

### Quantitative predictions based on HRT and non-HRT bioindicators

The data of bioreactor A and bioreactor B were used for training and testing the regression models independently. Due to the unbalanced ratio of HRT 8 days (14 samples with 26%) and HRT 2 days (40 samples with 74%), we also created models using balanced training datasets. The artificially balanced datasets were created based on the HRT class information and using SMOTE implemented in the R package UBL (v0.0.6) [100]. The balanced datasets had 52 and 48 samples for HRT 2 days and 8 days, respectively. For the process parameters to be predicted, four training datasets were considered: only with samples from bioreactor A, only with samples from bioreactor B and the balanced version of these two datasets. Initially, three algorithms including linear regression, support vector machine with radial kernel and random forest for regression (implemented in R package ranger, v0.12.1) [101] were employed as a heuristic approach to evaluate their predictive performance based on the metric root mean square error. The training and benchmarking processes were performed using the R package mlr (v2.18.0) [102]. All algorithms were validated using a 5-fold cross-validation approach. We selected the algorithm presenting better overall prediction performance and trained it with another round of 5-fold cross-validation. After, the random forest regression analysis was used to predict the process parameters specified as concentrations of lactate, C4, C6 and C8, and productivities as well as yields of C4, C6 and C8 (experiment summary and metadata table in Additional file 10). Alpha diversity metrics (i.e., observed ASV counts) was also considered a parameter in the quantitative prediction. Here, the relevance of the different ASVs to the prediction was determined by the residual sum of squares (IncNodePurity, default in randomForest) for the regressions. Explained variance (% Var. explained in R) was used to measure the model performance on the training set [97]. We predicted the accuracy by measuring how well the features can explain the variance of these process parameters on the test set [98]. The hyperparameters of random forest trained models (e.g., number of trees) were tuned heuristically during cross-validation.

We performed the quantitative prediction by applying a two-step regression analysis with 5-fold cross-validation (workflow in Additional file 1: Fig. S18). First, HRT bioindicators were used to predict the data of different process parameters in the sampling intervals of 0–50 days and 141–211 days. Data of all samples in the four HRT phases were considered controls. Relative abundance dataset of bioreactor A was used as training set and that of bioreactor B was used as test set and vice versa. Next, considering community assembly caused by time, we determined the ASVs (non-HRT bioindicators) that could predict the numeric values of each process parameter, using data of samples in the intervals of 0–50 days and 141–211 days. For each process parameter, we started with computing the feature importance of all ASVs and further selected the 15 top-rated ASVs as the bioindicators of this non-HRT parameter. Datasets of bioreactors A and B were independently used for training and testing. As controls, we used the non-HRT bioindicators of each parameter to predict the corresponding data of all samples in the four HRT phases. The final set of ASVs presented in HRT bioindicators and not in non-HRT bioindicators were considered HRT bioindicators irrespective of time.

### Evaluation of prediction accuracy

When in both training sets the HRT bioindicators and non-HRT bioindicators explained more than 80% of the variance in a process parameter, we proceeded only with those parameters. To compare the predicted and measured values for these process parameters, we considered the following performance metrics for reflecting the error of the model in predicting consecutive data: RRMSE, cutoff < 10%; R squared, slope and intercept of the least squares line of best fit. The final values of RRMSE were averaged among the 100 random forest replicates, with four ASVs for HRT bioindicators and five for non-HRT bioindicators randomly sampled at each replicate.

## Supplementary Information

**Additional file 1: Figure S1.** Gas production of bioreactors. **Figure S2.** Biomass production of bioreactors and correlation analysis. **Figure S3.** Microbial community composition profiles of bioreactors. **Figure S4.** Alpha diversity metrics of bioreactor communities. **Figure S5.** Predictive performance of three machine learning algorithms using HRT bioindicators. **Figure S6.** Predictive performance of three machine learning algorithms using non-HRT bioindicators for considering community assembly caused by time. **Figure S7.** Prediction results of C6 and C8 productivities using non-HRT bioindicators for considering community assembly caused by time. **Figure S8.** Prediction results of C6 and C8 productivities for all samples in the four HRT phases using HRT bioindicators. **Figure S9.** Prediction results of C6 and C8 productivities for all samples in the four HRT phases using non-HRT bioindicators for considering community assembly caused by time. **Figure S10.** Random forest feature importance of A-HRT bioindicators and B-HRT bioindicators used to predict C6 and C8 productivities. **Figure S11.** Random forest feature importance of the non-HRT bioindicators used to predict C6 and C8 productivities. **Figure S12.** Metabolic pathways involved in converting lactate and xylan to *n*-caproate and *n*-caprylate. **Figure S13.** Correlation network of environmental factors, process performance and microbial community. **Figure S14.** Prediction results of C6 and C8 productivities for all samples in the four HRT phases using the four ASVs of HRT bioindicators irrespective of time. **Figure S15.** Reducing HRT increases abundances of HRT bioindicators driving the catabolism of xylan and lactate to *n*-caproate and *n*-caprylate. **Figure S16.** Alpha rarefaction curves. **Figure S17.** Workflow of the random forest classification analysis. **Figure S18.** Workflow of a two-step random forest regression analysis. **Table S1.** Mean carboxylate yields (i.e., C mole product to substrate ratios) at HRTs of 8 days and 2 days (stable production period). **Table S2.** Explained variances of the training set in the regression-based prediction of process parameters using A-HRT bioindicators and B-HRT bioindicators. **Table S3.** Explained variances of the training set in the regression-based prediction of process parameters using non-HRT bioindicators for considering community assembly caused by time. **Table S4.** Growth medium used for the reactor operation. **Table S5.** Daily feeding of bioreactors A and B during the four HRT phases.

**Additional file 2.** Comparison of prediction accuracy of different algorithms using the mAML machine learning pipeline for classification.

**Additional file 3: Dataset S1.** MAGs taxonomy and genome metrics.

**Additional file 4: Dataset S2.** Functional annotations of xylose fermentation for MAGs with the same taxonomy as HRT bioindicators.

**Additional file 5: Dataset S3.** Functional annotations of chain elongation for MAGs with the same taxonomy as HRT bioindicators.

**Additional file 6: Dataset S4.** Functional annotations of xylose fermentation for all MAGs.

**Additional file 7: Dataset S5.** Functional annotations of chain elongation for all MAGs.

**Additional file 8.** ASV and taxonomy table.

**Additional file 9.** Gini scores of all ASVs in the classification-based prediction of HRT phases.

**Additional file 10.** Experimental summary and metadata table of all process parameters.

## Data Availability

All data described in this study are available in the paper or in the Supplementary material. Raw reads of amplicon sequencing data (ERR4158761 to ERR4158850) and metagenome sequencing data (ERR4183110 to ERR4183115) have been deposited in the European Nucleotide Archive (ENA) under study no. PRJEB38353. The MAGs are publicly available in ENA under the sample accession nos. ERS4594296 to ERS4594324.

## Abbreviations

ASVs: Amplicon sequence variants
C4: *n*-Butyrate
C6: *n*-Caproate
C8: *n*-Caprylate
CE: Chain elongation
FDR: False discovery rate
GTDB: Genome Taxonomy Database
HRT: Hydraulic retention time
MAGs: Metagenome-assembled genomes
NMDS: Nonmetric multidimensional scaling
OD: Optical density
PERMANOVA: Permutational multivariate analysis of variance
RRMSE: Relative root mean square error
SMOTE: Synthetic Minority Oversampling Technique
