# Supplementary material for: Machine learning-assisted identification of bioindicators predicts medium-chain carboxylate production performance of an anaerobic mixed culture
Source: Microbiome. 2022 Mar 25;10:48. doi: 10.1186/s40168-021-01219-2 (PMC8952268; doi:10.1186/s40168-021-01219-2)
Supplement: Supplementary file 2 — Additional file 1: Figure S1. Gas production of bioreactors. Figure S2. Biomass production of bioreactors and correlation analysis. Figure S3. Microbial community composition profiles of bioreactors. Figure S4. Alpha diversity metrics of bioreactor communities. Figure S5. Predictive performance of three machine learning algorithms using HRT bioindicators. Figure S6. Predictive performance of three machine learning algorithms using non-HRT bioindicators for considering community assembly caused by time. Figure S7. Prediction results of C6 and C8 productivities using non-HRT bioindicators for considering community assembly caused by time. Figure S8. Prediction results of C6 and C8 productivities for all samples in the four HRT phases using HRT bioindicators. Figure S9. Prediction results of C6 and C8 productivities for all samples in the four HRT phases using non-HRT bioindicators for considering community assembly caused by time. Figure S10. Random forest feature importance of A-HRT bioindicators and B-HRT bioindicators used to predict C6 and C8 productivities. Figure S11. Random forest feature importance of the non-HRT bioindicators used to predict C6 and C8 productivities. Figure S12. Metabolic pathways involved in converting lactate and xylan to n-caproate and n-caprylate. Figure S13. Correlation network of environmental factors, process performance and microbial community. Figure S14. Prediction results of C6 and C8 productivities for all samples in the four HRT phases using the four ASVs of HRT bioindicators irrespective of time. Figure S15. Reducing HRT increases abundances of HRT bioindicators driving the catabolism of xylan and lactate to n-caproate and n-caprylate. Figure S16. Alpha rarefaction curves. Figure S17. Workflow of the random forest classification analysis. Figure S18. Workflow of a two-step random forest regression analysis. Table S1. Mean carboxylate yields (i.e., C mole product to substrate ratios) at HRTs of 8 days and 2 days (stable productio [file 40168_2021_1219_MOESM2_ESM.pdf]

Machine learning-assisted identification of bioindicators predicts medium-chain carboxylate production performance of an anaerobic mixed culture

Additional File 1. Figures S1-S18 and Tables S1-S5

Bin Liu[1], Heike Sträuber[1], João Saraiva[1], Hauke Harms[1], Sandra Godinho Silva[2], Jonas Coelho Kasmanas[1,3,4], Sabine Kleinsteuber[1]* and Ulisses Nunes da Rocha[1]*

[1]Department of Environmental Microbiology, Helmholtz Centre for Environmental Research – UFZ, Leipzig, Germany

[2]Institute for Bioengineering and Biosciences, Department of Bioengineering, Instituto Superior Técnico Universidade de Lisboa, Lisbon, Portugal

[3]Institute of Mathematics and Computer Sciences, University of São Paulo, São Carlos, Brazil

[4]Department of Computer Science and Interdisciplinary Center of Bioinformatics, University of Leipzig, Leipzig, Germany

Corresponding authors

sabine.kleinsteuber@ufz.de / ulisses.rocha@ufz.de (ordered alphabetically according to last name).

*These authors contributed euqally to this work

Contents

Figure S1. Gas production of bioreactors. 3

Figure S2. Biomass production and correlation of bioreactors. 4

Figure S3. Microbial community composition profiles of bioreactors. 7

Figure S4. Alpha diversity metrics of bioreactor communities. 8

Figure S5. Predictive performance of three machine learning algorithms using HRT bioindicators. 9

Figure S6. Predictive performance of three machine learning algorithms using non-HRT bioindicators for considering community assembly caused by time. 10

Figure S7. Prediction results of C6 and C8 productivities using non-HRT bioindicators for considering community assembly caused by time. 11

Figure S8. Prediction results of C6 and C8 productivities for all samples in the four HRT phases using HRT bioindicators. 12

Figure S9. Prediction results of C6 and C8 productivities for all samples in the four HRT phases using non-HRT bioindicators for considering community assembly caused by time. 13

Figure S10. Random forest feature importance of A-HRT bioindicators and B-HRT bioindicators used to predict C6 and C8 productivities. 14

Figure S11. Random forest feature importance of the non-HRT bioindicators used to predict C6 and C8 productivities. 15

Figure S12. Metabolic pathways involved in converting lactate and xylan to *n*-caproate and *n*-caprylate. 17

Figure S13. Correlation network of environmental factors, process performance and microbial community. 18

Figure S14. Prediction results of C6 and C8 productivities for all samples in the four HRT phases using the four ASVs of HRT bioindicators irrespective of time. 19

Figure S15. Reducing HRT increases abundances of HRT bioindicators driving the catabolism of xylan and lactate to *n*-caproate and *n*-caprylate. 20

Figure S16. Alpha rarefaction curves. 22

Figure S17. Workflow of the random forest classification analysis. 23

Figure S18. Workflow of a two-step random forest regression analysis. 23

Table S1. Mean carboxylate yields (i.e. C mole product to substrate ratios) at HRTs of 8 days and 2 days (stable production period). 24

Table S2. Explained variances of the training set in the regression-based prediction of process parameters using A-HRT bioindicators and B-HRT bioindicators. 25

Table S3. Explained variances of the training set in the regression-based prediction of process parameters using non-HRT bioindicators for considering community assembly caused by time. 26

Table S4. Growth medium used for the reactor operation. 27

Table S5. Daily feeding of bioreactors A and B during the four HRT phases. 28

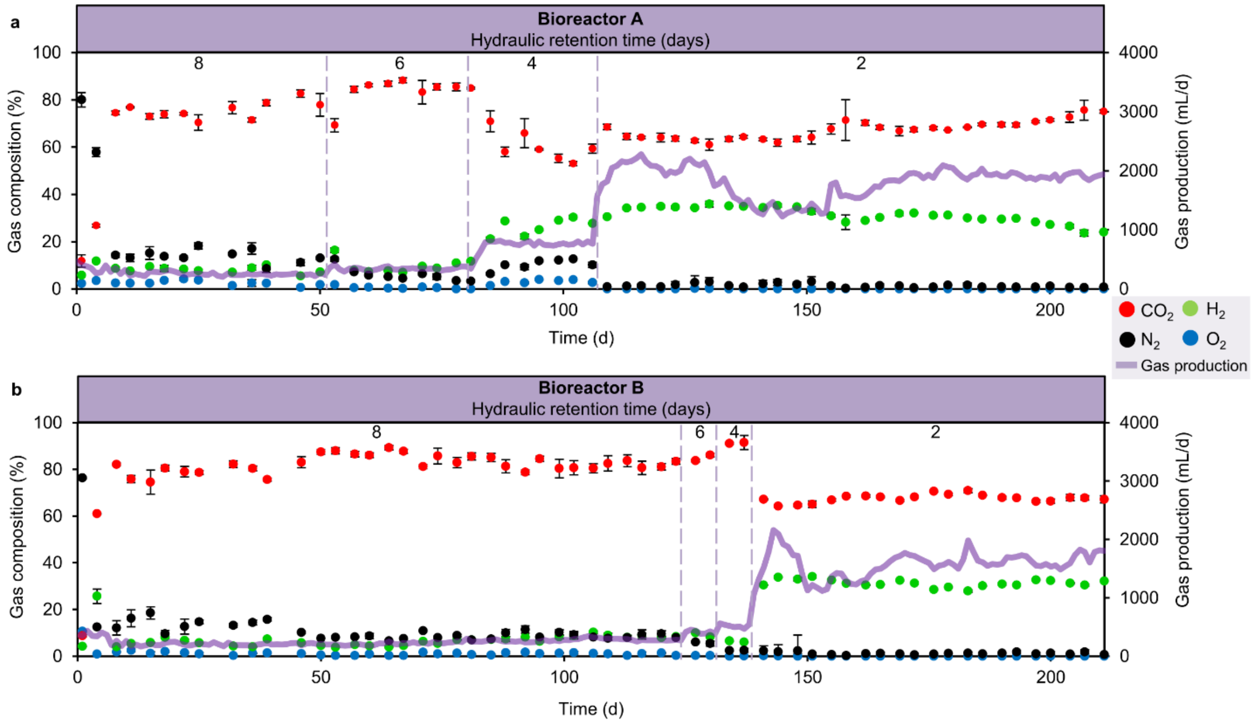

Figure S1. Gas production of bioreactors. Daily gas production and composition in bioreactors A (**a**) and B (**b**), respectively, during the four HRT phases. Error bars indicate the standard deviation.

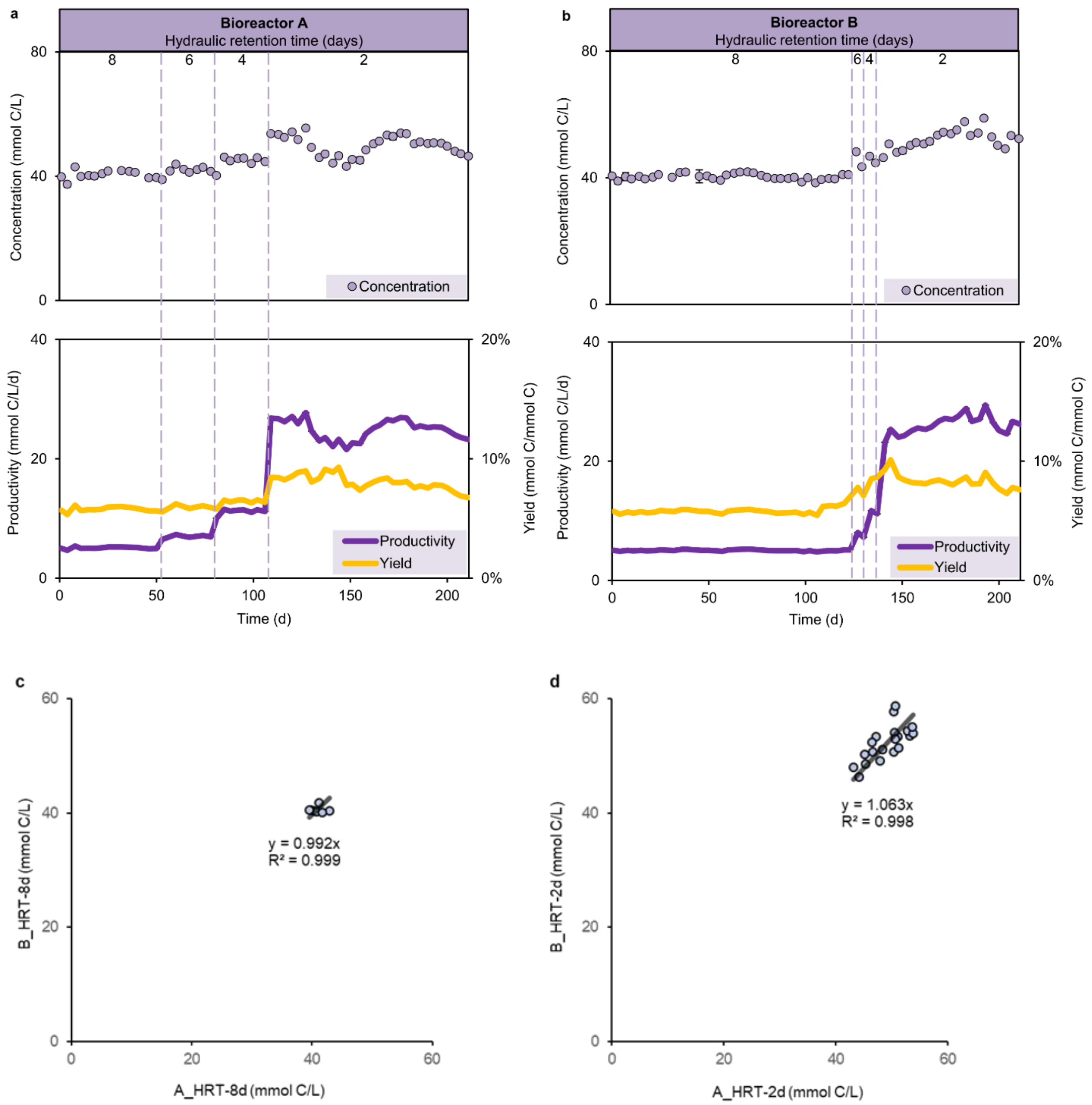

Figure S2. Biomass production and correlation of bioreactors. Cell concentration, biomass productivity and biomass yield in bioreactors A (**a**) and B (**b**) during the four HRT phases; and the correlations of cell concentration between bioreactors A and B at HRTs of 8 days (**c**) and 2 days (**d**) within the same time period. The carbon number of cell biomass was calculated by assuming an elemental biomass composition of $CH_{1.8}O_{0.5}N_{0.2}$ (molar mass = 24.6 g mol^{-1}). Error bars represent the standard deviation.

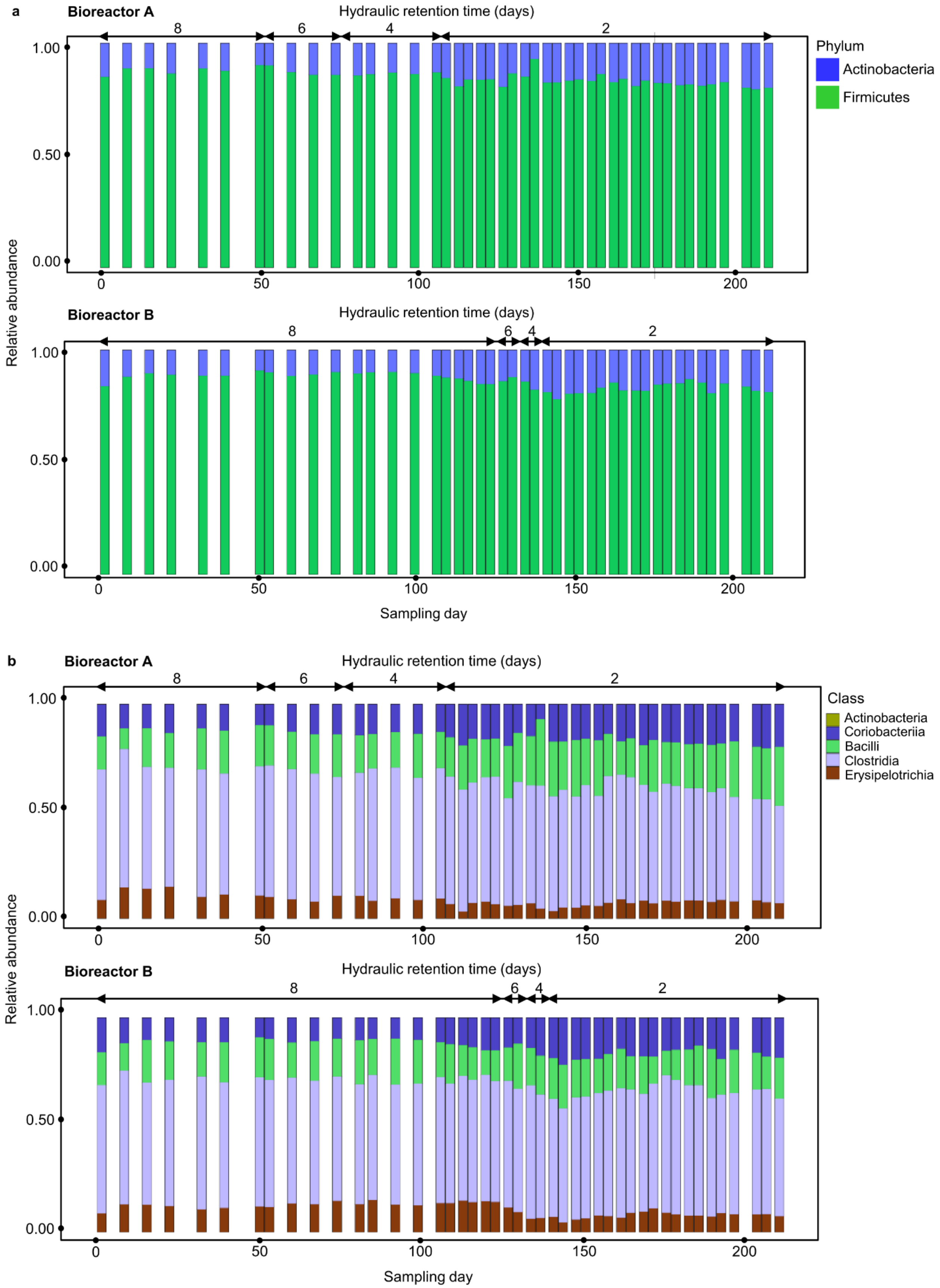
a
Bioreactor A
Hydraulic retention time (days)
8
6
4
2
1.00
0.50
0.00
0
50
100
150
200
Phylum
Actinobacteria
Firmicutes
Bioreactor B
Hydraulic retention time (days)
8
6
4
2
Relative abundance
Sampling day
b
Bioreactor A
Hydraulic retention time (days)
Class
Actinobacteria
Coriobacteriia
Bacilli
Clostridia
Erysipelotrichia
Bioreactor B
Relative abundance
Sampling day

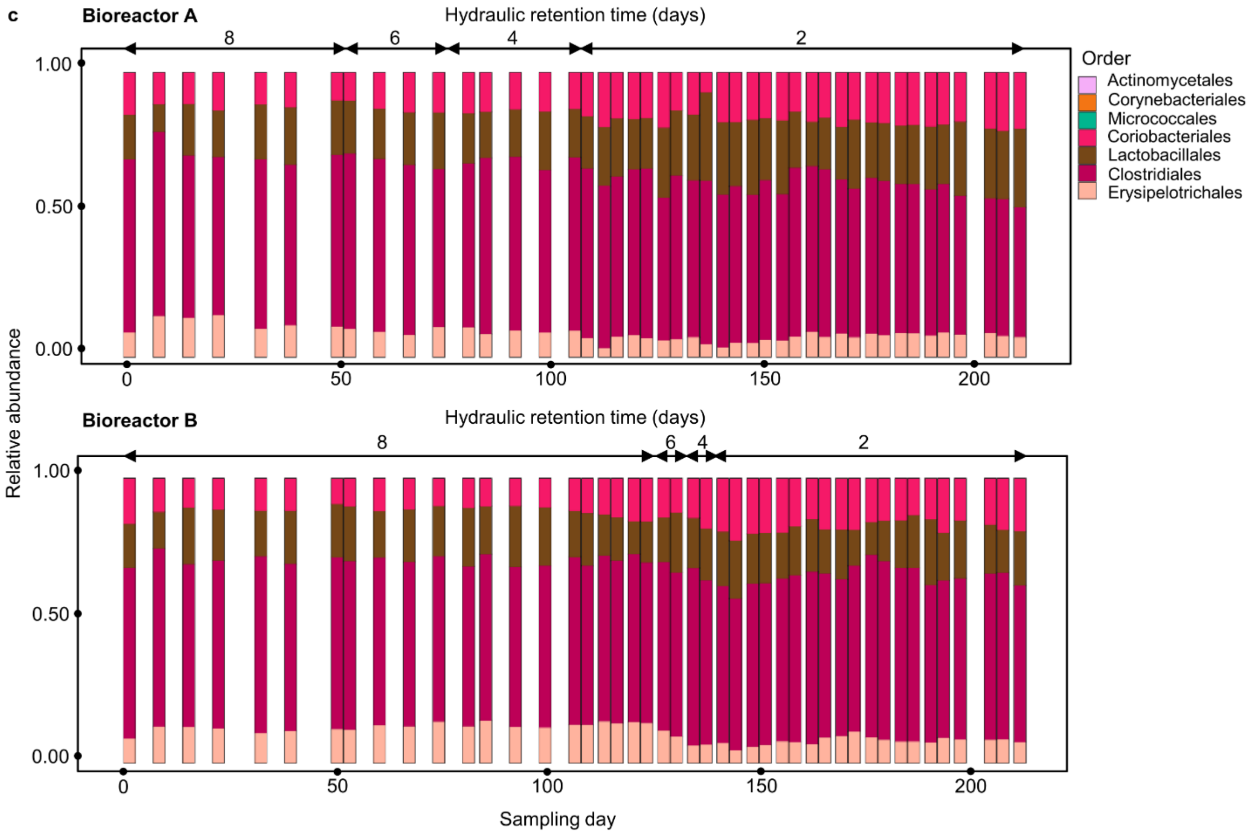
c
Bioreactor A
Hydraulic retention time (days)
8
6
4
2
1.00
0.50
0.00
0
50
100
150
200
Bioreactor B
Hydraulic retention time (days)
8
6
4
2
Relative abundance
Sampling day
Order
Actinomycetales
Corynebacteriales
Micrococcales
Coriobacteriales
Lactobacillales
Clostridiales
Erysipelotrichales

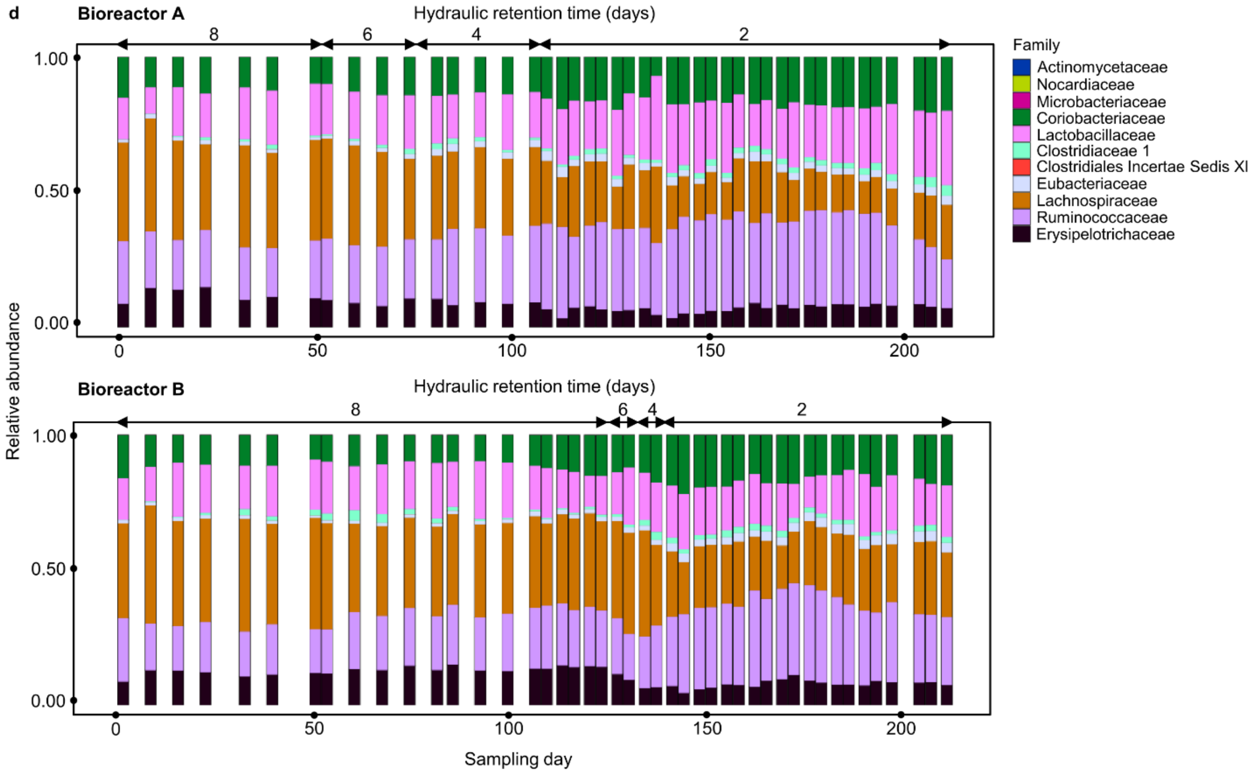
d
Bioreactor A
Hydraulic retention time (days)
8
6
4
2
1.00
0.50
0.00
0
50
100
150
200
Bioreactor B
Hydraulic retention time (days)
8
6
4
2
Relative abundance
Sampling day
Family
Actinomycetaceae
Nocardiaceae
Microbacteriaceae
Coriobacteriaceae
Lactobacillaceae
Clostridiaceae 1
Clostridiales Incertae Sedis XI
Eubacteriaceae
Lachnospiraceae
Ruminococcaceae
Erysipelotrichaceae

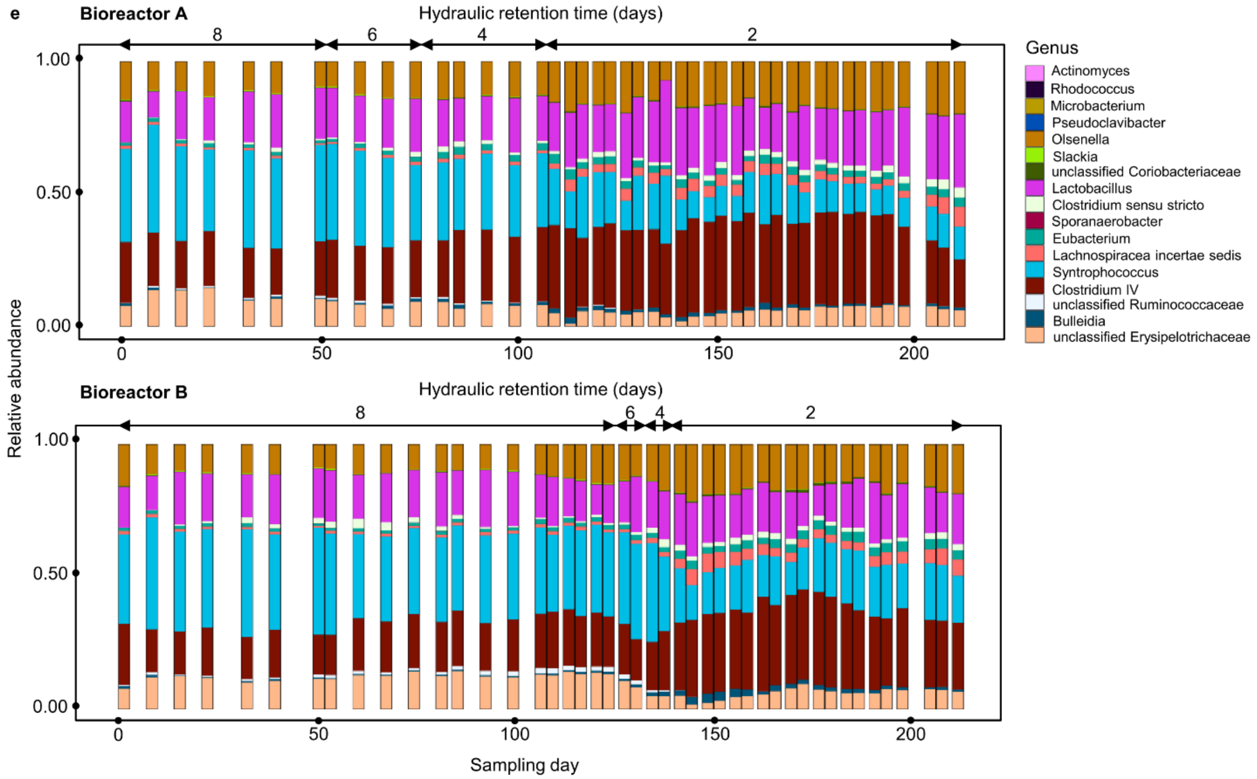

Figure S3. Microbial community composition profiles of bioreactors. Based on amplicon sequencing of 16S rRNA genes, the taxonomic classification of amplicon sequence variants (ASVs) was categorised at the phylum (**a**), class (**b**), order (**c**), family (**d**) and genus (**e**) levels.

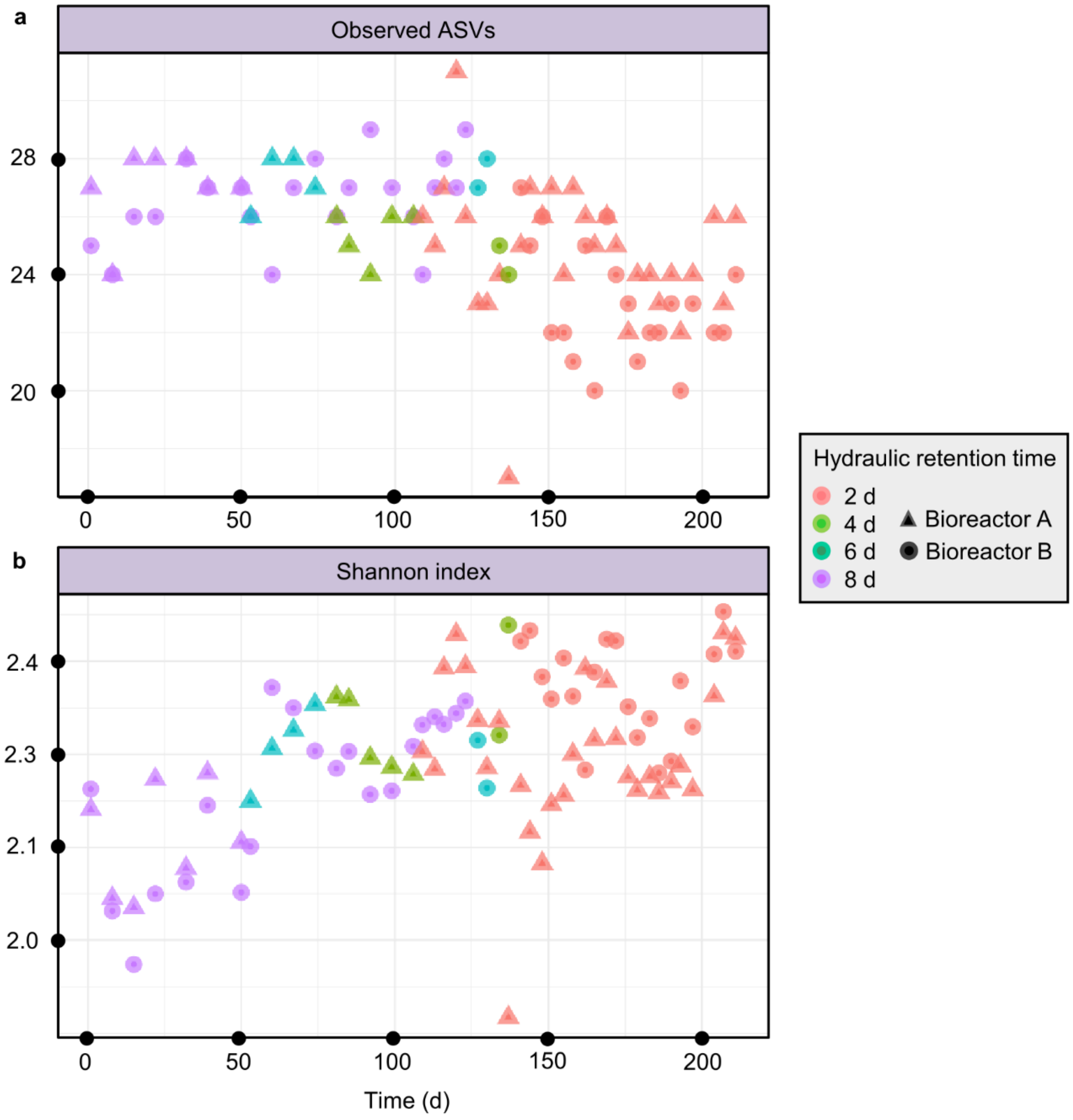

Figure S4. Alpha diversity metrics of bioreactor communities. Based on the relative abundance of ASVs, we calculated the alpha diversity represented by observed ASV counts (**a**) and Shannon index (**b**).

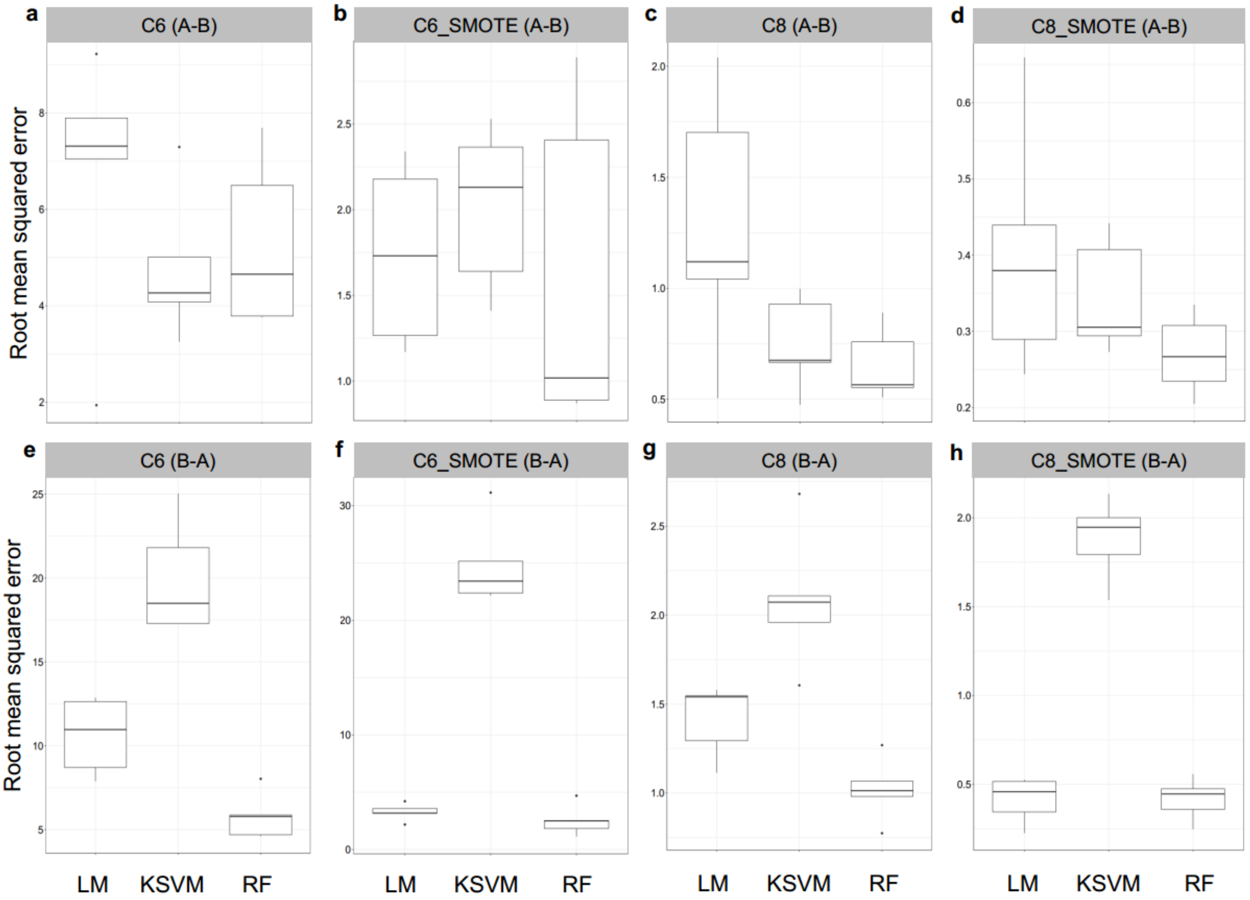

Figure S5. Predictive performance of three machine learning algorithms using HRT bioindicators. a,b, prediction of C6 productivity using the relative abundance data of bioreactor A for training the models and data of bioreactor B for testing, without (**a**) and with SMOTE (**b**) for balancing the oversampled datasets. **c,d,** prediction of C8 productivity using the relative abundance data of bioreactor A for training the models and data of bioreactor B for testing, without (**c**) and with SMOTE (**d**) for balancing. **e,f,** prediction of C6 productivity using the relative abundance data of bioreactor B for training the models and data of bioreactor A for testing, without (**e**) and with SMOTE (**f**) for balancing. **g,h,** prediction of C8 productivity using the relative abundance data of bioreactor B for training the models and data of bioreactor A for testing, without (**g**) and with SMOTE (**h**) for balancing. LM, linear regression; KSVM, support vector machine with radial kernel; RF, random forest regression.

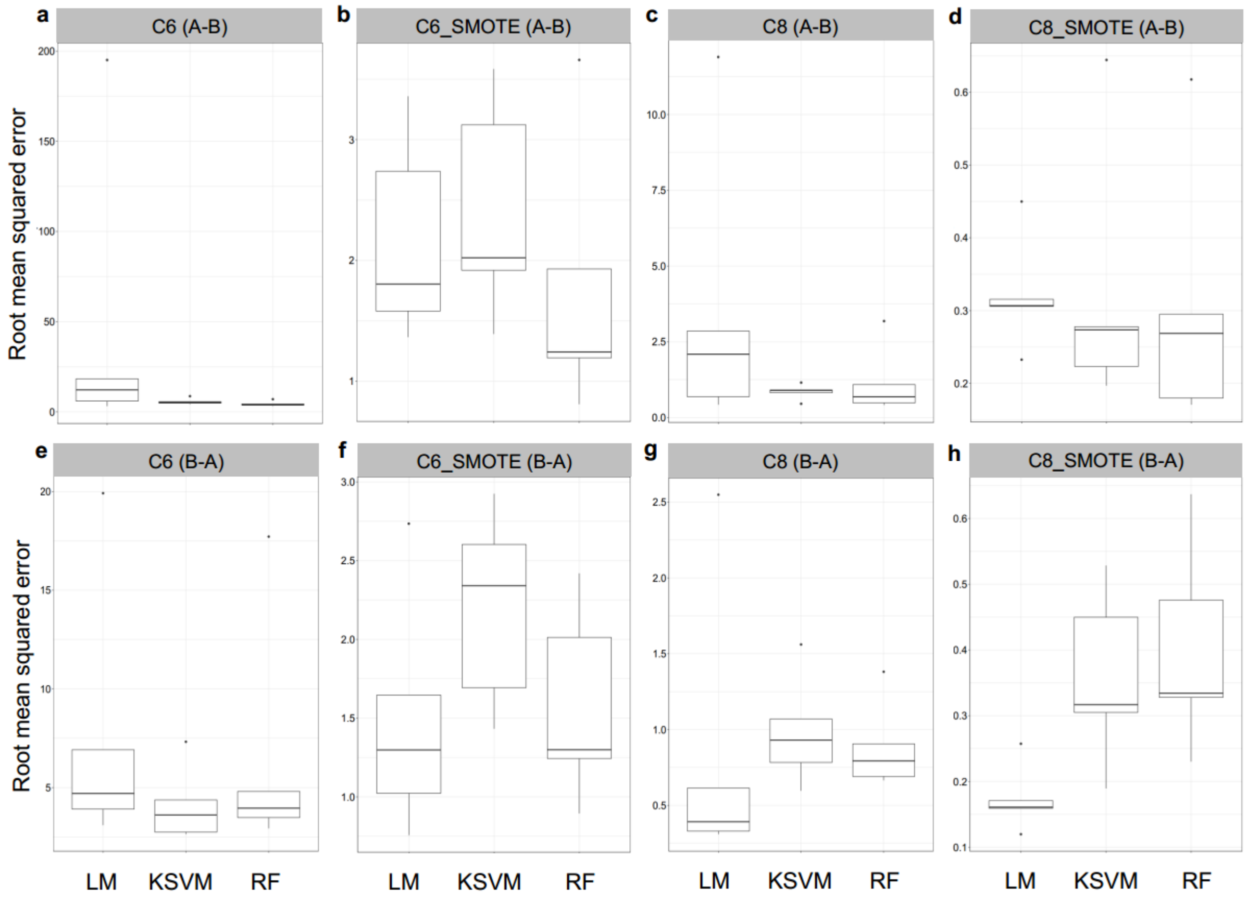

Figure S6. Predictive performance of three machine learning algorithms using non-HRT bioindicators for considering community assembly caused by time. a,b, prediction of C6 productivity using the relative abundance data of bioreactor A for training the models and data of bioreactor B for testing, without (**a**) and with SMOTE (**b**) for balancing the oversampled datasets. **c,d,** prediction of C8 productivity using the relative abundance data of bioreactor A for training the models and data of bioreactor B for testing, without (**c**) and with SMOTE (**d**) for balancing. **e,f,** prediction of C6 productivity using the relative abundance data of bioreactor B for training the models and data of bioreactor A for testing, without (**e**) and with SMOTE (**f**) for balancing. **g,h,** prediction of C8 productivity using the relative abundance data of bioreactor B for training the models and data of bioreactor A for testing, without (**g**) and with SMOTE (**h**) for balancing. LM, linear regression; KSVM, support vector machine with radial kernel; RF, random forest regression.

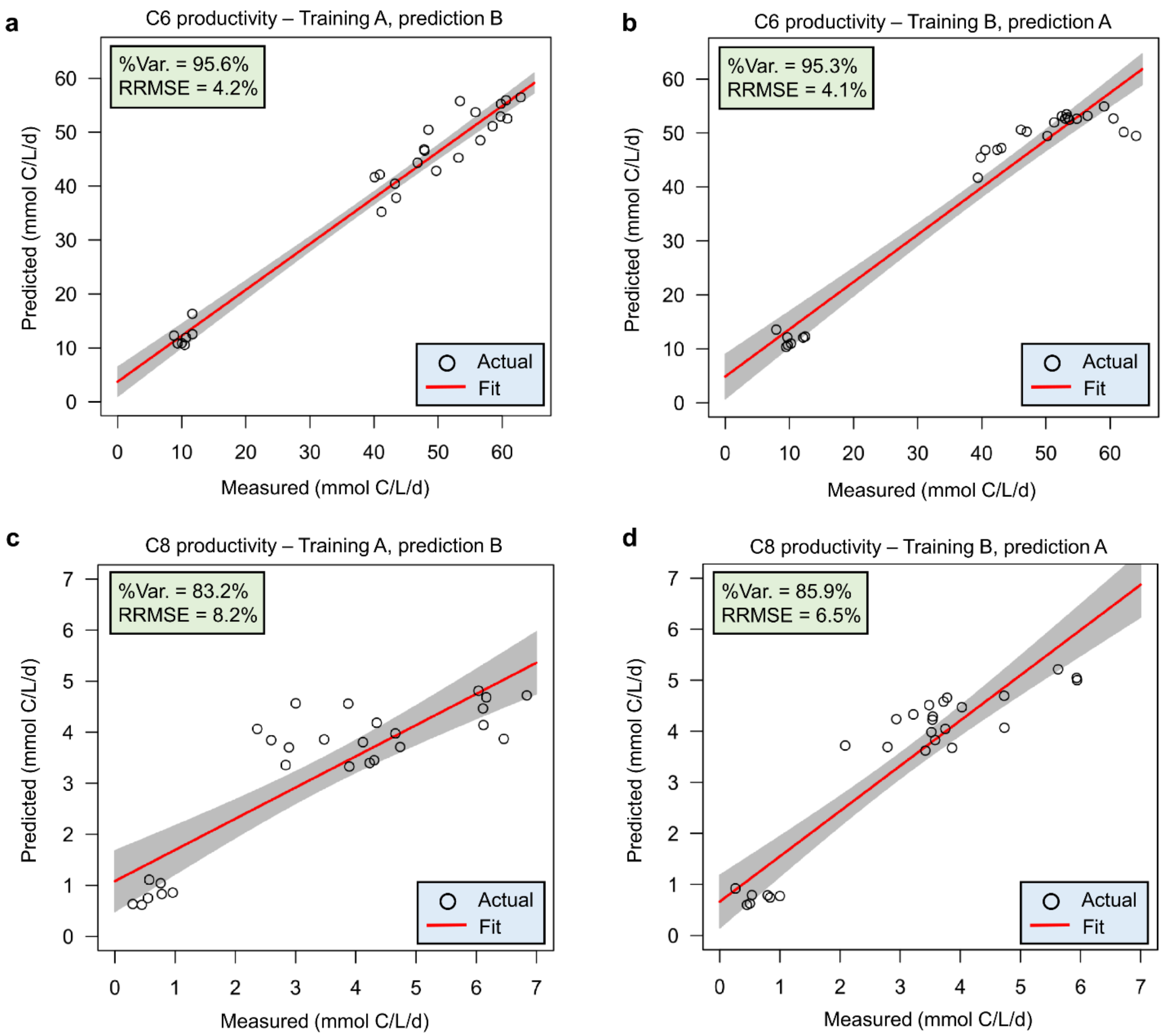

Figure S7. Prediction results of C6 and C8 productivities using non-HRT bioindicators for considering community assembly caused by time. a,b, Prediction performance using C6 productivity bioindicators of bioreactors A and B. **c,d,** Prediction performance using C8 productivity bioindicators of bioreactors A and B. Results in **a** and **c** were obtained by using the relative abundance data of bioreactor A for training the models and data of bioreactor B for testing. Results using the data of bioreactor B for training and bioreactor A for testing are shown in **b** and **d**. %Var., explains the variance (%) in C6/C8 productivity of the training set. RRMSE, relative root mean square error.

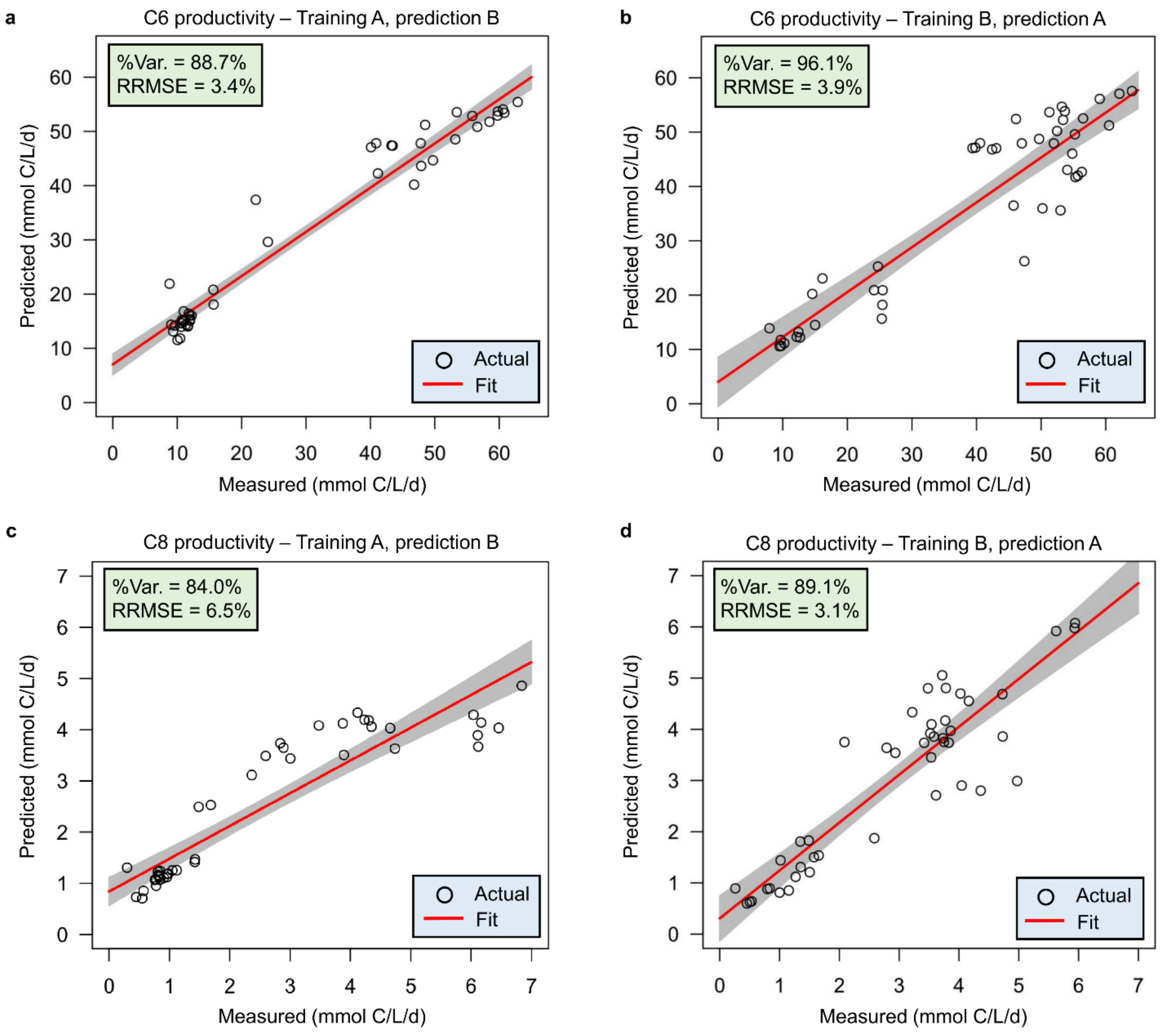

Figure S8. Prediction results of C6 and C8 productivities for all samples in the four HRT phases using HRT bioindicators. a,b, Prediction performance of C6 productivity. **c,d,** Prediction performance of C8 productivity. Results in **a** and **c** were obtained by using the relative abundance data of bioreactor A for training the models and data of bioreactor B for testing. Results using data of bioreactor B for training and bioreactor A for testing are shown in **b** and **d**. %Var., explains the variance (%) in C6/C8 productivity of the training set. RRMSE, relative root mean square error.

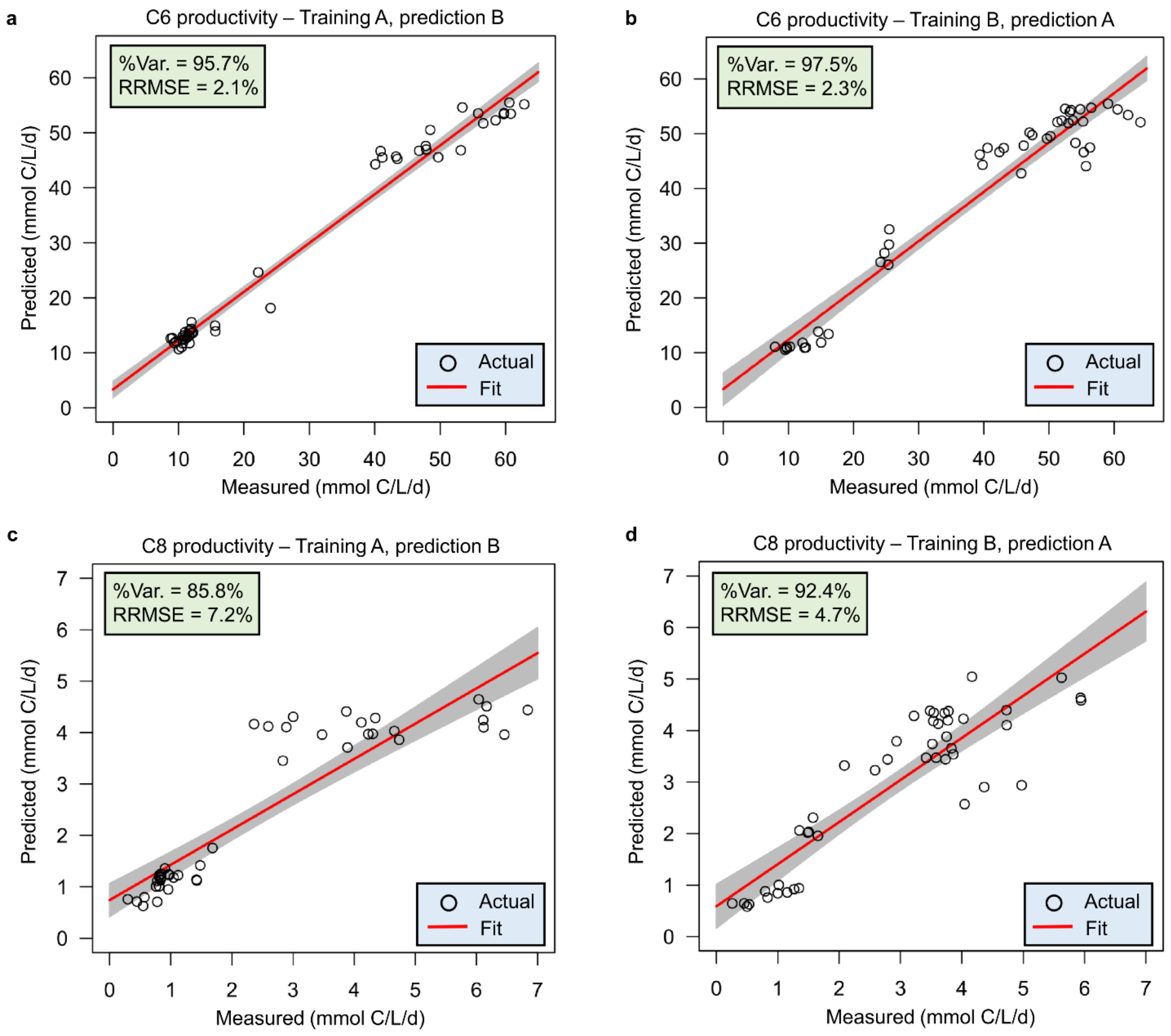

Figure S9. Prediction results of C6 and C8 productivities for all samples in the four HRT phases using non-HRT bioindicators for considering community assembly caused by time. **a,b,** Prediction performance using C6 productivity bioindicators of bioreactors A and B. **c,d,** Prediction performance using C8 productivity bioindicators of bioreactors A and B. Results in **a** and **c** were obtained by using the relative abundance data of bioreactor A for training the models and data of bioreactor B for testing. Results using the data of bioreactor B for training and bioreactor A for testing are shown in **b** and **d**. %Var., explains the variance (%) in C6/C8 productivity of the training set. RRMSE, relative root mean square error.

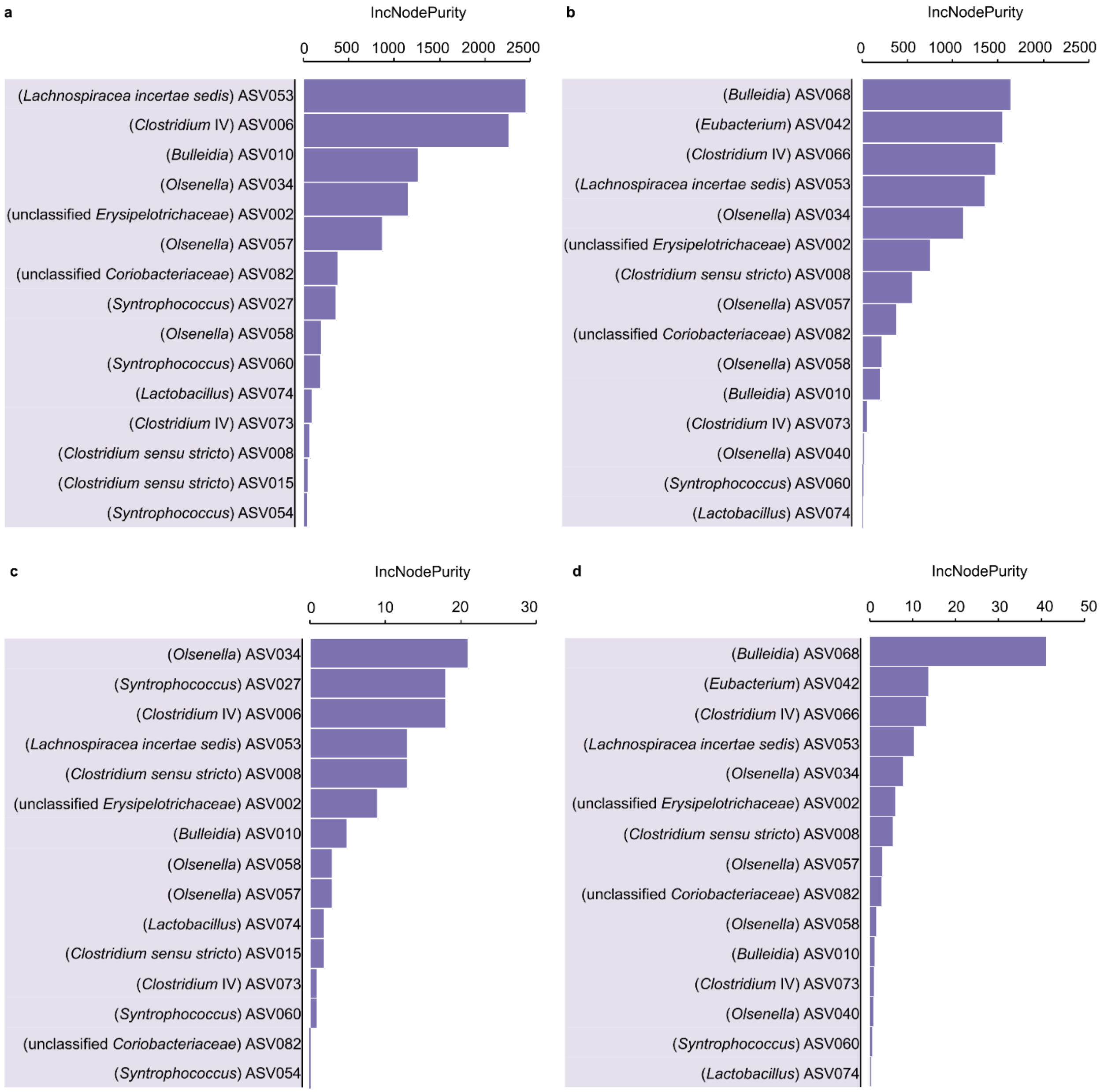

Figure S10. Random forest feature importance of A-HRT bioindicators and B-HRT bioindicators used to predict C6 and C8 productivities. a, Feature importance of A-HRT bioindicators in the prediction of C6 productivity. **b,** Feature importance of B-HRT bioindicators in the prediction of C6 productivity. **c,** Feature importance of A-HRT bioindicators in the prediction of C8 productivity. **d,** Feature importance of B-HRT bioindicators in the prediction of C8 productivity. IncNodePurity, residual sum of squares.

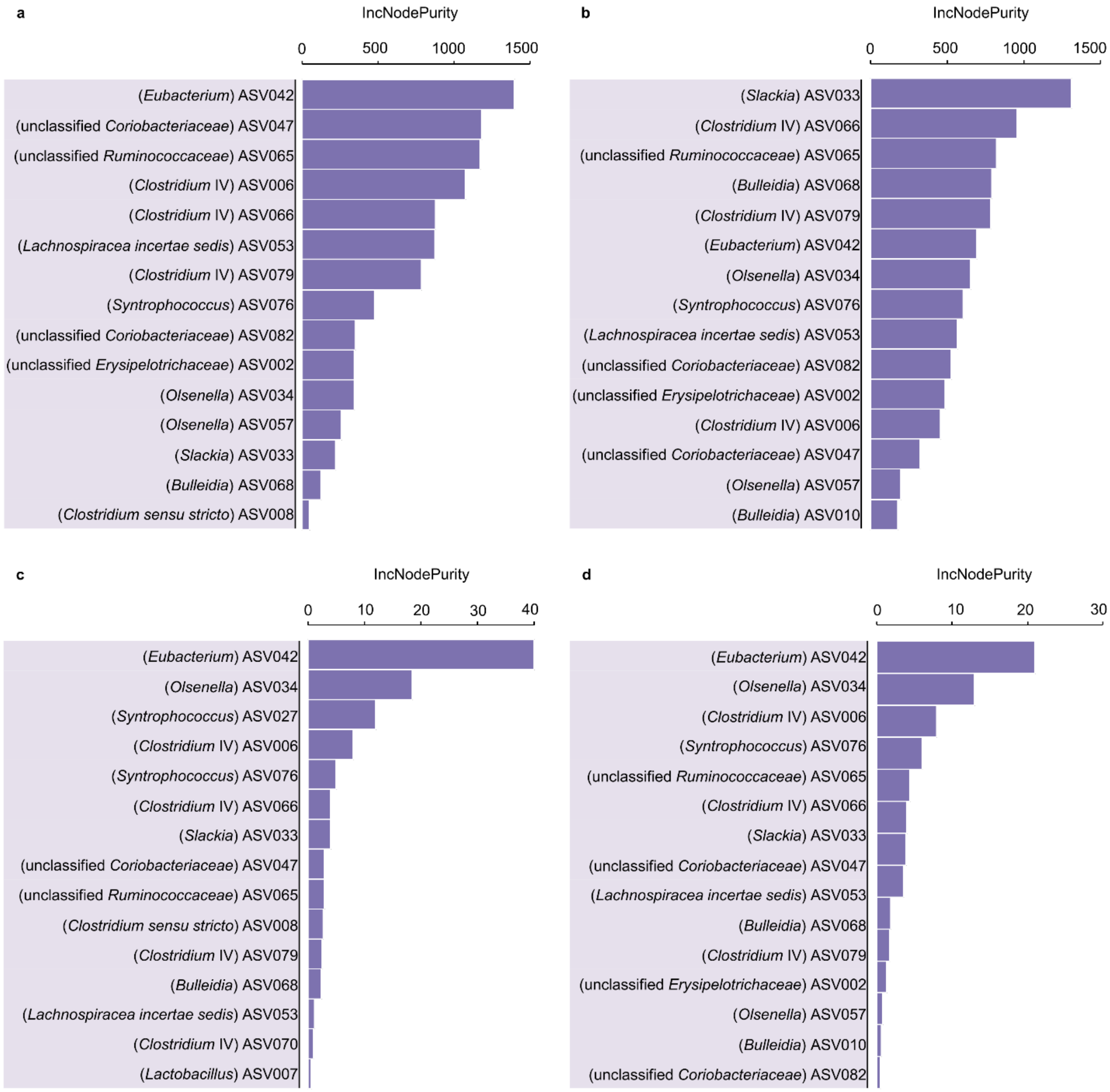

Figure S11. Random forest feature importance of the non-HRT bioindicators used to predict C6 and C8 productivities. a,b, The feature importance of C6 productivity bioindicators of bioreactors A and B. **c,d,** The feature importance C8 productivity bioindicators of bioreactors A and B. Relative abundance data of bioreactor A were used as training set and that of bioreactor B as test set (**a,c**); while data of bioreactor B for training and that of bioreactor A for testing (**b,d**). IncNodePurity, residual sum of squares.

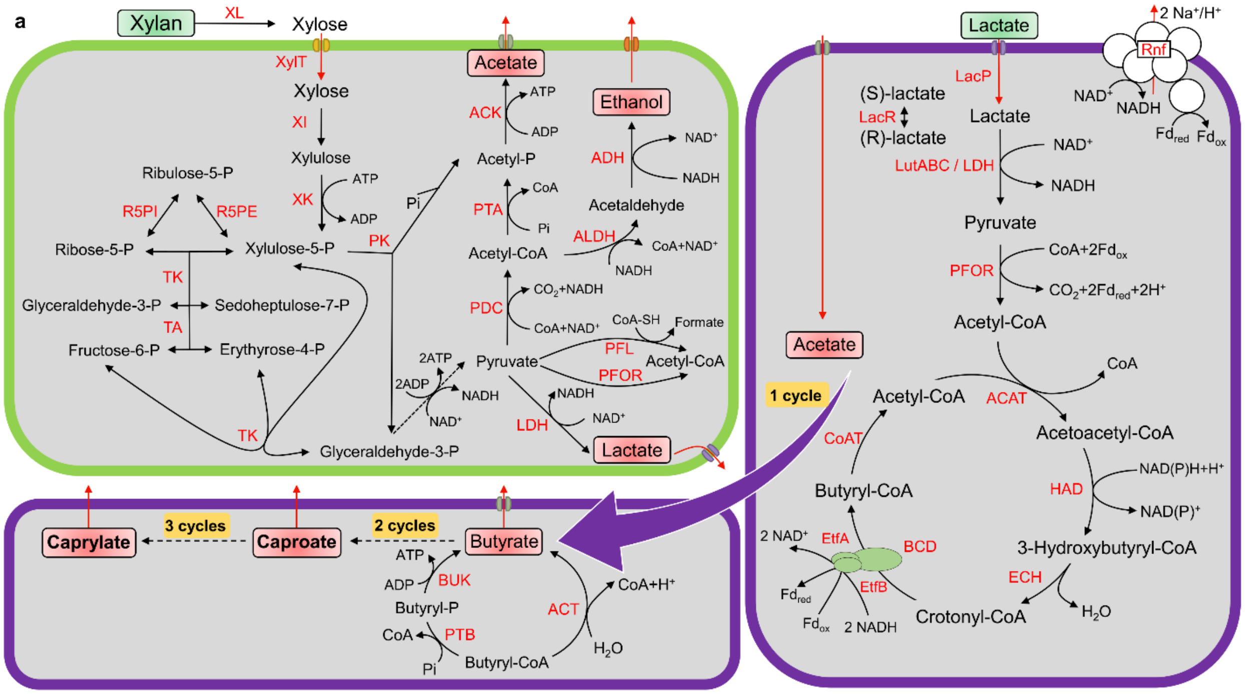

b

Predicted function	No.	Enzyme abbreviation	EC number	Enzyme
Xylan degradation	1	XL	3.2.1.8	xylanase
Xylose utilization	2	XylT	7.5.2.10;7.5.2.13	xylose transporter
	3	XI	5.3.1.5	xylose isomerase
	4	XK	2.7.1.17	xylulose kinase
	5	R5PE	5.1.3.1	ribulose-5-phosphoate epimerase
	6	R5PI	5.3.1.6	ribose-5-phosphate isomerase
	7	TA	2.2.1.2	transaldolase
	8	TK	2.2.1.1	transketolase
	9	PK	4.1.2.9	D-xylulose 5-phosphate phosphoketolase
Pyruvate transformation	10	PDC	1.2.4.1;2.3.1.2;1.8.1.4	pyruvate dehydrogenase complex
	11	PFOR	1.2.7.-	pyruvate flavodoxin oxidoreductase
	12	PFL	2.3.1.54	formate C-acetyltransferase (pyruvate formate lyase)
	13	ALDH	1.2.1.10	acetaldehyde dehydrogenase
	14	ADH	1.1.1.1	alcohol dehydrogenase
	15	PTA	2.3.1.8	phosphate acetyltransferase
	16	ACK	2.7.2.1	acetate kinase
	17	LDH	1.1.1.27;1.1.1.28	lactate dehydrogenase
	18	PTB	2.3.1.19	phosphate butyryltransferase
	19	BUK	2.7.2.7	butyrate kinase

c

Predicted function	No.	Enzyme abbreviation	EC number	Enzyme
Lactate utilization	1	LacR	5.1.2.1	lactate racemase
	2	LacP	2.A.14	lactate permease
	3	LutABC		lactate utilization protein ABC
Acetyl-CoA formation	4	LDH	1.1.1.27	lactate dehydrogenase
	5	PFOR	1.2.7.1	pyruvate ferredoxin oxidoreductase
Reverse β-oxidation	6	ACAT	2.3.1.9, 2.3.1.16	acetyl-CoA acetyltransferase
	7	HAD	1.1.1.157, 1.1.1.35	3-hydroxyacyl-CoA dehydrogenase
	8	ECH	4.2.1.150, 4.2.1.55	enoyl-CoA hydratase
	9	BCD	1.3.8.1	butyryl-CoA dehydrogenase
	10	EtfAB		electron transfer flavoprotein A,B
	11	CoAT	2.8.3.-	butyryl-CoA:acetate CoA-transferase
	12	ACT	3.1.2.20	acyl-CoA thioesterase
Energy conservation	13	RnfABCDEG	7.1.1.1	energy-converting NADH: ferredoxin oxidoreductase

Figure S12. Metabolic pathways involved in converting lactate and xylan to *n*-caproate and *n*-caprylate. a, The pathways involved in the processes of anaerobic mixed culture fermentation include hydrolysis of xylan (more than 95% xylooligosaccharides), fermentation of xylose and chain elongation with lactate as electron donor. The enzyme abbreviations are provided in red letters next to the pathways (solid lines). **b,** Enzymes of the predicted functions related to xylan hydrolysis, xylose fermentation and pyruvate transformation. **c,** Enzymes of the predicted functions related to chain elongation with lactate as electron donor. Dashed lines represent multi-enzyme reactions between the two indicated molecules. In **(a)**, "cycle" refers to the reverse β-oxidation cycle. The functional annotation of metagenome-assembled genomes (MAGs) with the same taxonomy as HRT bioindicators can be found in Additional file 3: Dataset S2 (for xylan hydrolysis and xylose fermentation) and Additional file 4: Dataset S3 (for chain elongation). The functional annotation of all MAGs can be found in Additional file 5: Dataset S4 (for xylan hydrolysis and xylose fermentation) and Additional file 6: Dataset S5 (for chain elongation).

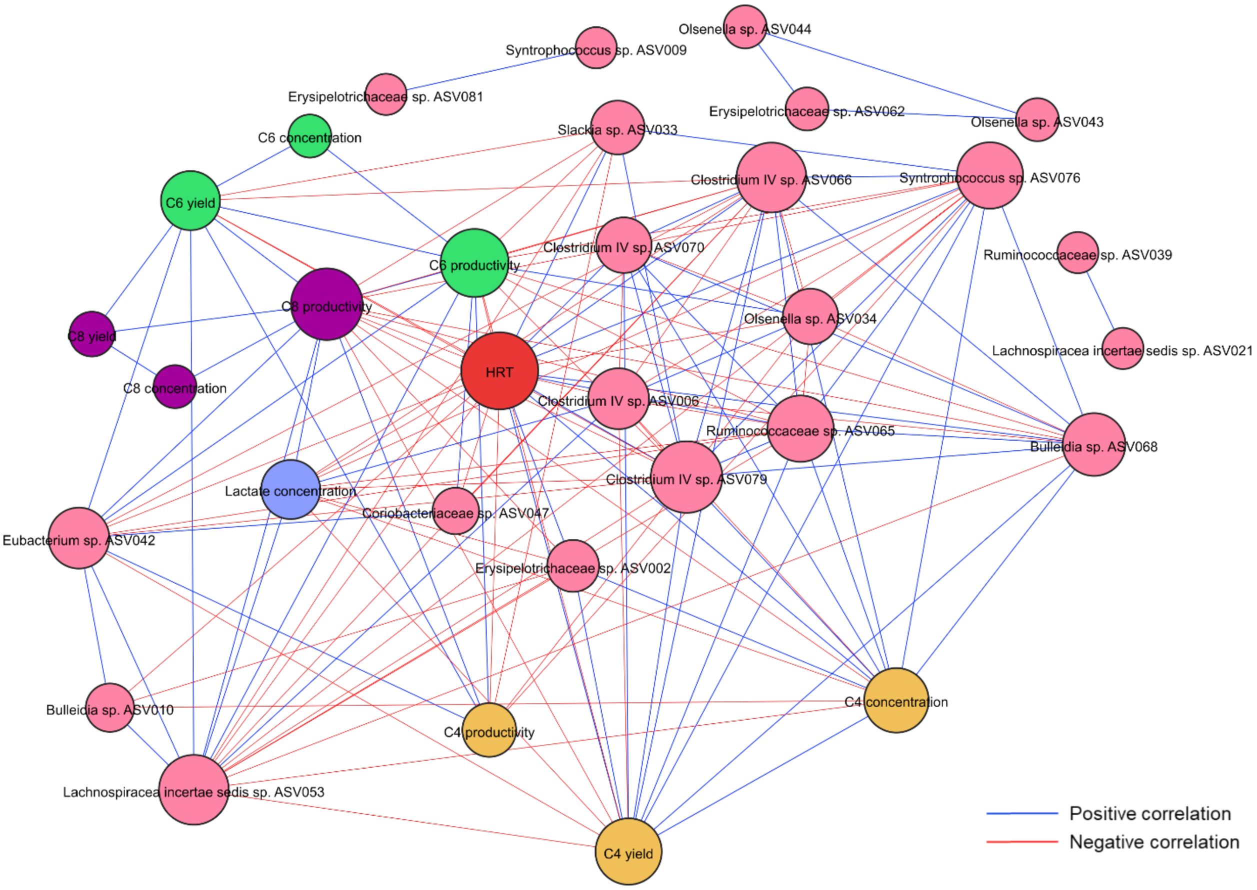

Figure S13. Correlation network of environmental factors, process performance and microbial community. Edges indicate the Spearman coefficient > 0.7 for positive correlations (blue edges) and < 0.7 for negative correlations (red edges). Node size was scaled to represent its degree of connectedness. Here, the environmental factors represent controlled operational parameters with only reducing the HRT, and the process performance refers to the concentration, productivity and yield of the target products. C4, *n*-butyrate; C6, *n*-caproate; C8, *n*-caprylate.

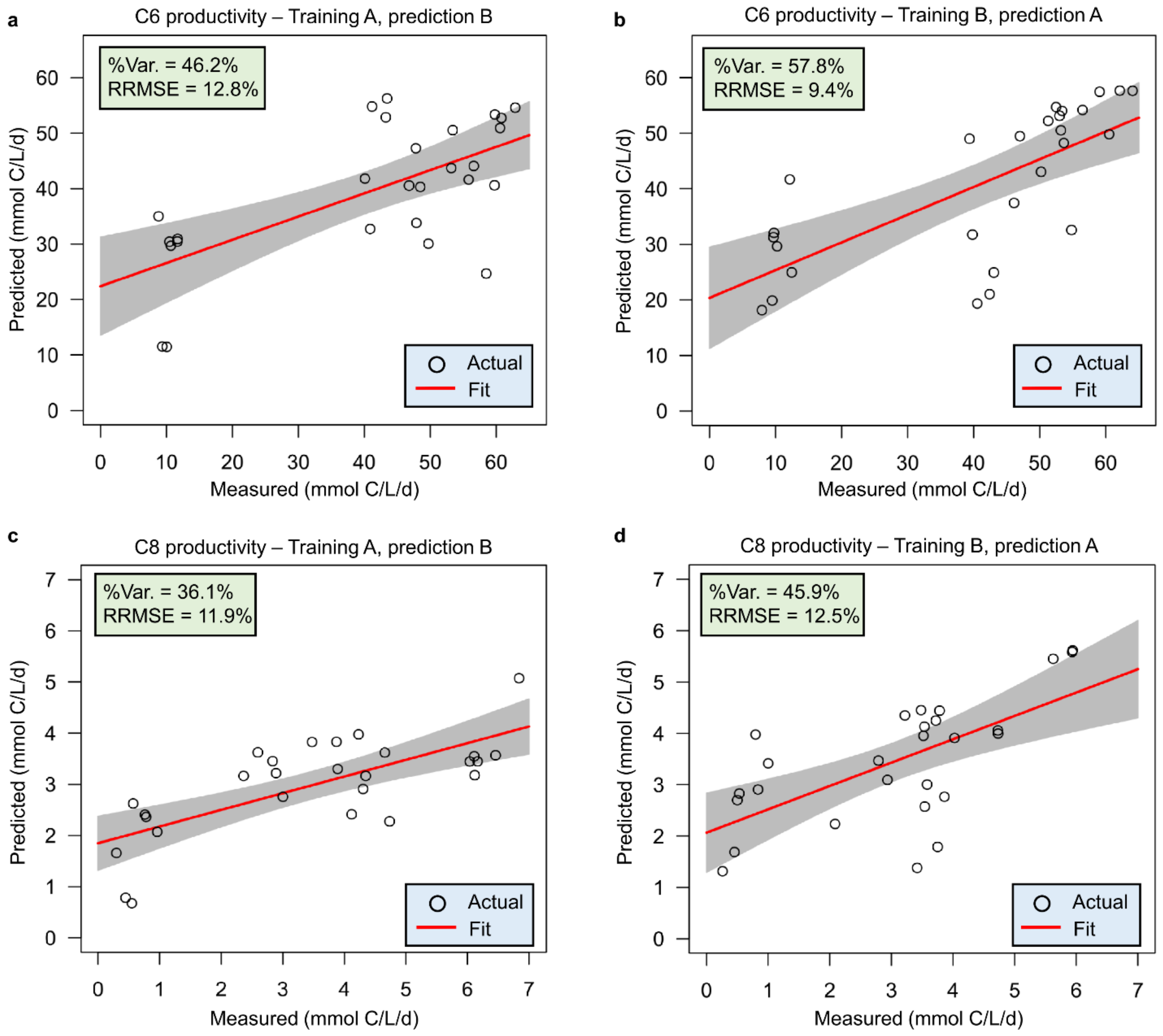

Figure S14. Prediction results of C6 and C8 productivities for all samples in the four HRT phases using the four ASVs of HRT bioindicators irrespective of time. a,b, Prediction performance of C6 productivity. **c,d,** Prediction performance of C8 productivity. Results in **a** and **c** were obtained by using the relative abundance data of bioreactor A for training the models and data of bioreactor B for testing. Results using the data of bioreactor B for training and bioreactor A for testing are shown in **b** and **d**. %Var., explains the variance (%) in C6/C8 productivity of the training set. RRMSE, relative root mean square error.

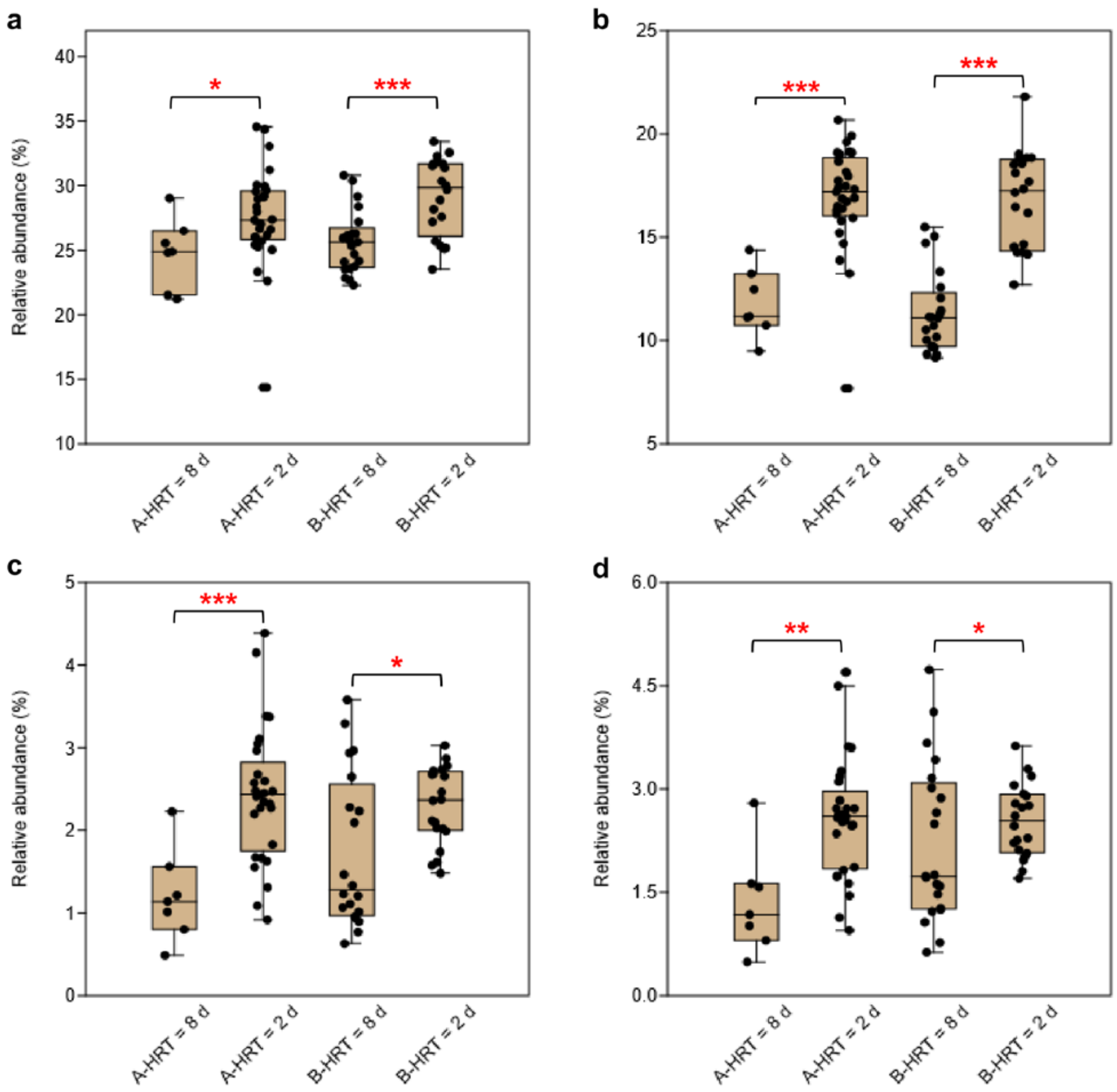

Figure S15. Reducing HRT increases abundances of HRT bioindicators driving the catabolism of xylan and lactate to *n*-caproate and *n*-caprylate. These catabolic steps were categorised into four main functions of the anaerobic mixed culture fermentation. **a,** Hydrolysis of xylan. Relevant HRT bioindicators are *Olsenella* sp. ASV034, *Olsenella* sp. ASV057, *Olsenella* sp. ASV058, unclassified *Erysipelotrichaceae* sp. ASV002, *Bulleidia* sp. ASV010, *Lachnospiracea incertae sedis* ASV053, *Syntrophococcus* sp. ASV060 and *Clostridium* IV sp.

ASV073. **b,** Xylose fermentation producing acetate and lactate. Relevant HRT bioindicators are *Olsenella* sp. ASV034, *Olsenella* sp. ASV057, *Olsenella* sp. ASV058 and *Lactobacillus* sp. ASV074. **c,** Butyrate formation from lactate and acetate. Relevant HRT bioindicators are *Syntrophococcus* sp. ASV060 and *Clostridium sensu stricto* sp. ASV008. **d,** Chain elongation with lactate as electron donor producing *n*-butyrate, *n*-caproate and *n*-caprylate. Relevant HRT bioindicators are *Syntrophococcus* sp. ASV060, *Clostridium* IV sp. ASV073 and *Clostridium sensu stricto* sp. ASV008. The rarefied ASV abundances were calculated using 16S rRNA amplicon sequencing data. Boxes represent the interquartile range between the 25th and 75th percentiles, respectively, the line inside denote the median value, and asterisks indicate significant different mean values (adjusted P*** < 0.001 < ** < 0.01 < * < 0.05), which is tested by permutational Student's *t*-test with 9,999 simulations. On the horizontal axis, A and B stand for bioreactors A and B, respectively.

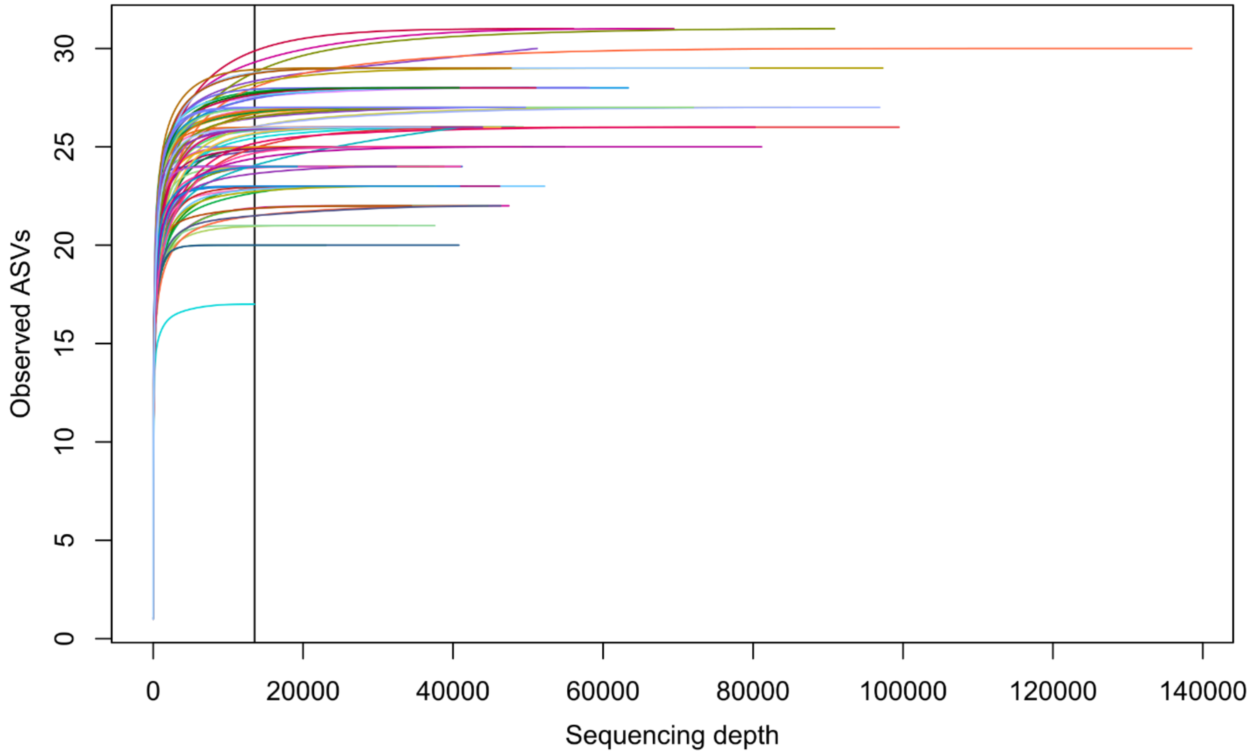

Figure S16. Alpha rarefaction curves. ASVs of all samples were rarefied to an equal sequencing depth of 13,518 reads. Colours represent the different samples.

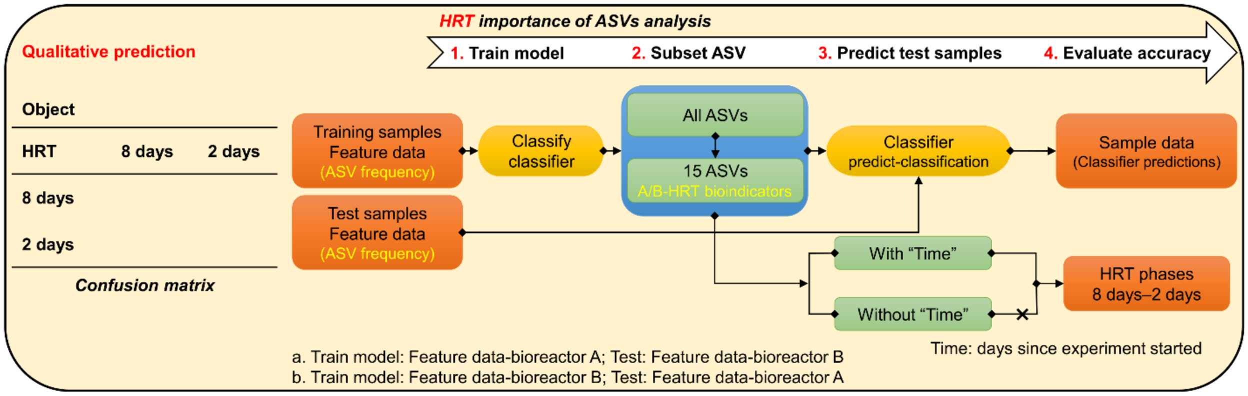

Figure S17. Workflow of the random forest classification analysis. The qualitative prediction of HRT phases of 8 days and 2 days. Letters in yellow show the input (ASV frequency) and output (A/B-HRT bioindicators) of the model.

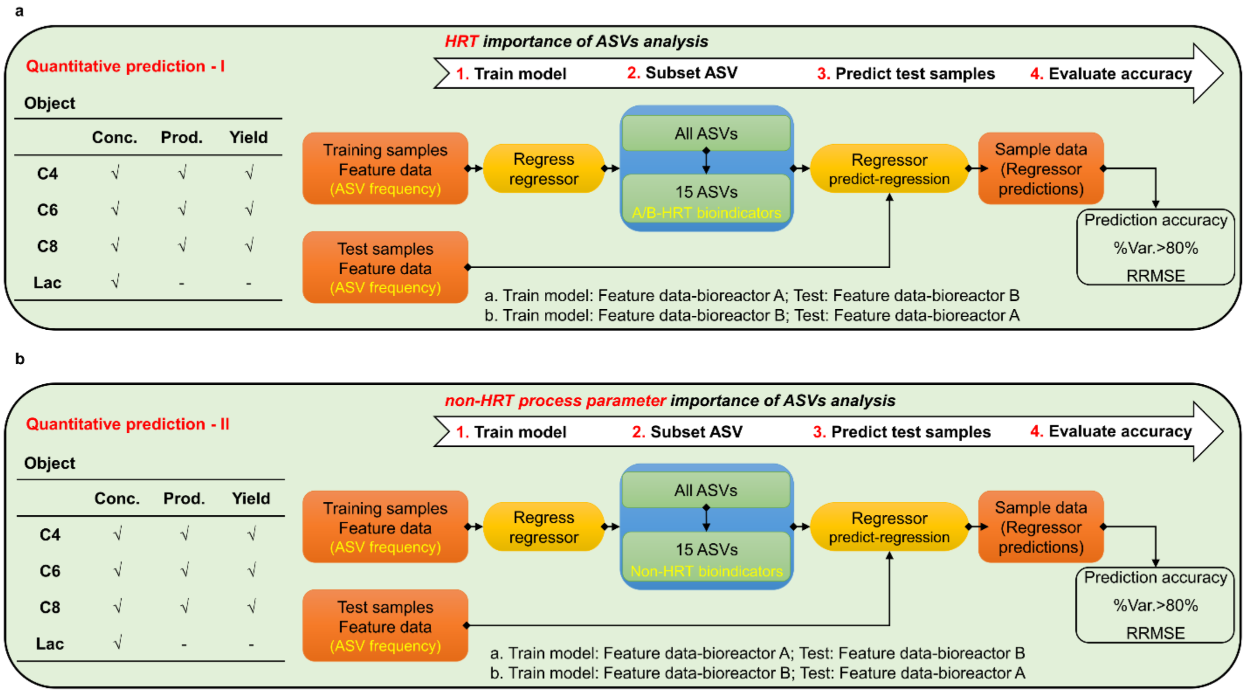

Figure S18. Workflow of a two-step random forest regression analysis. a, The first step of quantitative predictions using HRT bioindicators. **b,** The second step of quantitative predictions using non-HRT bioindicators for considering community assembly caused by time. Lac, lactate; Conc., concentration; Prod., productivity; %Var., explains the variance (%) in process parameters of the training set. RRMSE, relative root mean square error.

Table S1. Mean carboxylate yields (i.e. C mole product to substrate ratios) at HRTs of 8 days and 2 days (stable production period).

Bioreactor	HRT (d)	Duration (d)	C4 (mmol C/mmol C)	C6 (mmol C/mmol C)	C8 (mmol C/mmol C)
A	8	0-51	39.1	11.6	0.7
	2	162-211	26.8	16.9	1.3
B	8	0-123	39.6	12.3	0.9
	2	193-211	22.3	18.7	2.0

Table S2. Explained variances of the training set in the regression-based prediction of process parameters using A-HRT bioindicators and B-HRT bioindicators. Features (ASVs) explaining more than 80% of the variance in a process parameter are indicated in green. A and B stand for bioreactors A and B, respectively.

Training set	Predicted variable		Set of ASVs	Number of ASVs	Explained variance (%)
Bioreactor A	concentration	C4	ASV_all	71	89.37 ± 0.48
			A-HRT bioindicators	15	87.81 ± 0.54
		C6	ASV_all	71	67.66 ± 0.84
			A-HRT bioindicators	15	68.06 ± 0.84
		C8	ASV_all	71	58.43 ± 0.91
			A-HRT bioindicators	15	59.30 ± 1.00
		Lactate	ASV_all	71	77.30 ± 0.79
			A-HRT bioindicators	15	78.20 ± 0.77
	productivity	C4	ASV_all	71	88.61 ± 0.41
			A-HRT bioindicators	15	88.94 ± 0.35
		C6	ASV_all	71	93.80 ± 0.45
			A-HRT bioindicators	15	95.87 ± 0.23
		C8	ASV_all	71	80.91 ± 0.78
			A-HRT bioindicators	15	86.62 ± 0.51
	yield	C4	ASV_all	71	85.39 ± 0.58
			A-HRT bioindicators	15	80.81 ± 0.73
		C6	ASV_all	71	73.81 ± 0.64
			A-HRT bioindicators	15	54.62 ± 0.98
		C8	ASV_all	71	60.50 ± 0.81
			A-HRT bioindicators	15	56.84 ± 0.92
Bioreactor B	concentration	C4	ASV_all	71	79.49 ± 0.49
			B-HRT bioindicators	15	78.61 ± 0.63
		C6	ASV_all	71	73.94 ± 0.54
			B-HRT bioindicators	15	77.11 ± 0.50
		C8	ASV_all	71	62.37 ± 0.75
			B-HRT bioindicators	15	63.64 ± 0.83
		Lactate	ASV_all	71	68.64 ± 0.85
			B-HRT bioindicators	15	72.02 ± 0.66
	productivity	C4	ASV_all	71	65.90 ± 0.63
			B-HRT bioindicators	15	67.91 ± 0.58
		C6	ASV_all	71	93.92 ± 0.36
			B-HRT bioindicators	15	93.44 ± 0.37
		C8	ASV_all	71	84.37 ± 0.68
			B-HRT bioindicators	15	85.65 ± 0.58
	yield	C4	ASV_all	71	68.67 ± 0.82
			B-HRT bioindicators	15	70.99 ± 0.49
		C6	ASV_all	71	73.57 ± 0.54
			B-HRT bioindicators	15	78.17 ± 0.41
		C8	ASV_all	71	65.75 ± 0.83
			B-HRT bioindicators	15	67.19 ± 0.93

Note. Explained variances of the variable observed ASVs were lower than 60% in all cases.

Table S3. Explained variances of the training set in the regression-based prediction of process parameters using non-HRT bioindicators for considering community assembly caused by time. Features (ASVs) explaining more than 80% of the variance in a process parameter are indicated in green. A and B stand for bioreactors A and B, respectively.

Training set	Predicted variable		Set of ASVs	Number of ASVs	Explained variance (%)
Bioreactor A	concentration	C4	ASV_all	71	89.37 ± 0.48
			A-C4c_bioindicators	15	91.69 ± 0.25
		C6	ASV_all	71	67.66 ± 0.84
			A-C6c_bioindicators	15	70.72 ± 0.57
		C8	ASV_all	71	58.43 ± 0.91
			A-C8c_bioindicators	15	63.95 ± 0.54
		Lactate	ASV_all	71	77.30 ± 0.79
			A-LACc_bioindicators	15	79.38 ± 0.53
	productivity	C4	ASV_all	71	88.61 ± 0.41
			A-C4p_bioindicators	15	88.83 ± 0.26
		C6	ASV_all	71	93.80 ± 0.45
			A-C6p_bioindicators	15	95.64 ± 0.20
		C8	ASV_all	71	80.91 ± 0.78
			A-C8p_bioindicators	15	83.20 ± 0.48
	yield	C4	ASV_all	71	85.39 ± 0.58
			A-C4y_bioindicators	15	87.07 ± 0.36
		C6	ASV_all	71	73.81 ± 0.64
			A-C6y_bioindicators	15	74.73 ± 0.40
		C8	ASV_all	71	60.50 ± 0.81
			A-C8y_bioindicators	15	61.85 ± 0.66
Bioreactor B	concentration	C4	ASV_all	71	79.49 ± 0.49
			B-C4c_bioindicators	15	81.50 ± 0.33
		C6	ASV_all	71	73.94 ± 0.54
			B-C6c_bioindicators	15	75.15 ± 0.36
		C8	ASV_all	71	62.37 ± 0.75
			B-C8c_bioindicators	15	64.35 ± 0.57
		Lactate	ASV_all	71	68.64 ± 0.85
			B-LACc_bioindicators	15	71.16 ± 0.51
	productivity	C4	ASV_all	71	65.90 ± 0.63
			B-C4p_bioindicators	15	67.90 ± 0.41
		C6	ASV_all	71	93.92 ± 0.36
			B-C6p_bioindicators	15	95.25 ± 0.24
		C8	ASV_all	71	84.37 ± 0.68
			B-C8p_bioindicators	15	85.90 ± 0.53
	yield	C4	ASV_all	71	68.67 ± 0.82
			B-C4y_bioindicators	15	71.17 ± 0.46
		C6	ASV_all	71	73.57 ± 0.54
			B-C6y_bioindicators	15	74.70 ± 0.35
		C8	ASV_all	71	65.75 ± 0.83
			B-C8y_bioindicators	15	67.84 ± 0.52

Note. Explained variances of the variable observed ASVs were lower than 60% in all cases.

Table S4. Growth medium used for the reactor operation. The medium was anoxic by flushing with nitrogen and adjusted to pH 5.5 with 1 M sodium hydroxide solution.

Concentrations (all components were prepared completely sterile):			
0.054 g/L	$MgCl_2 \times 6\ H_2O$		
0.065 g/L	$CaCl_2 \times 2\ H_2O$		
0.474 g/L	NH_4Cl		
0.5 g/L	Yeast extract		
10.94 g/L	KH_2PO_4		
20.83 g/L	K_2HPO_4		
0.032 g/L	Na_2CO_3		
0.03 g/L	Cysteine-HCl		
1 mL/L	Trace element solution	1 mL/L	Vitamin solution
$FeCl_2 \times 4\ H_2O$	1.5 g/L	Biotin	20 mg/L
$CuCl_2 \times 2\ H_2O$	2 mg/L	Folic acid	20 mg/L
$CoCl_2 \times 6\ H_2O$	190 mg/L	Pyridoxine	100 mg/L
$MnCl_2$	100 mg/L	Thiamine	50 mg/L
$Na_2MoO_4 \times 2\ H_2O$	36 mg/L	Riboflavin	50 mg/L
$NiCl_2 \times 6\ H_2O$	24 mg/L	Nicotinic acid	50 mg/L
$Na_2WO_4 \times 2\ H_2O$	20 mg/L	Calcium pantothenate	50 mg/L
$Na_2SeO_3 \times 5\ H_2O$	3 mg/L	Vitamin B_{12}	20 mg/L
$ZnCl_2$	70 mg/L	*p*-Amino benzoic acid	80 mg/L
H_3BO_3	6 mg/L	Lipoic acid	50 mg/L

Table S5. Daily feeding of bioreactors A and B during the four HRT phases.

Bioreactor	HRT (d)	Duration (d)	Daily medium feeding				Daily effluent withdrawing (mL)
			Lactate (g)	Xylan (g)	Mineral medium (mL)	Deionised anoxic water (mL)	
A	8	0-51	1.47	1.25	11	114	125
	6	52-80	1.96	1.67	15	152	167
	4	81-106	2.94	2.50	23	227	250
	2	107-211	5.88	5.00	45	455	500
B	8	0-123	1.47	1.25	11	114	125
	6	124-130	1.96	1.67	15	152	167
	4	131-137	2.94	2.50	23	227	250
	2	138-211	5.88	5.00	45	455	500
